# Synthesis and Biological Evaluation of RGD-Conjugated MEK1/2 Kinase Inhibitors for Integrin-Targeted Cancer Therapy

**DOI:** 10.3390/molecules181113957

**Published:** 2013-11-12

**Authors:** Xiaoxiao Li, Jianjun Hou, Chao Wang, Xinjie Liu, Hongyan He, Ping Xu, Zhenjun Yang, Zili Chen, Yun Wu, Lihe Zhang

**Affiliations:** 1 Department of Chemistry, Renmin University of China, Beijing 100872, China; E-Mail: lixiaoxiao.2005@163.com; 2 State Key Laboratory of Natural and Biomimetic Drugs, School of Pharmaceutical Sciences, Peking University, Beijing, 100191, China; E-Mails: hou618@163.com (J.H.); sky10311@163.com (C.W.); xuer_liu@yeah.net (X.L.); hehy@pku.edu.cn (H.H.); pingxu@bjmu.edu.cn (P.X.); yangzj@bjmu.edu.cn (Z.Y.); zdszlh@bjmu.edu.cn (L.Z.)

**Keywords:** RGD-MEKI conjugate, MEK1/2 kinase inhibitor, PD0325901, RGD peptide, integrin α_v_β_3_ receptor, tumor-targeted drug delivery

## Abstract

Two novel series of RGD-MEKI conjugates derived from a MEK1/2 kinase inhibitor—PD0325901—have been developed for integrin receptor mediated anticancer therapy. The first series, alkoxylamine analog RGD-MEKI conjugates **9a**–**g** showed anti-proliferation activity in melanoma A375 cells by the same mechanism as that of PD0325901. PEGylation increased the IC_50_ value of **9f** three-fold in the A375 assay, and the multi-cRGD peptide cargo significantly improved the receptor specific anti-proliferation activity of **9g** in integrin-overexpressing U87 cells. In the second series, RGD-PD0325901 **13** exhibited significantly increased antitumor properties compared to the alkoxylamine analogs by both inhibition of the ERK pathway activity and DNA replication of the cancer cells. Furthermore, **13** displayed more potent anti-proliferation activity in the U87 assay than PD0325901 in a dose-dependent manner. All these data demonstrate that RGD-MEKI conjugates with an ester bond linkage enhanced anticancer efficacy with improved targeting capability toward integrin-overexpressing tumor cells.

## 1. Introduction

The RAS-RAF-MEK-ERK signaling cascade plays an important role in normal cell biological process, and is activated in many human tumors to mediate tumor progression and metastasis [1,2]. MEK1/2 kinases are downstream effectors of the ERK pathway. These dual-specificity protein kinases are considered as the important targets for new cancer drug development and have attracted much attention of medicinal chemists over the years [3,4,5,6]. PD0325901 is an oral active and selective second generation non-ATP-competitive MEK inhibitor. In the past decade, PD0325901 has shown great promise to treat advanced malignancies, such as melanoma, breast, lung (NSCLC), colon cancer, and papillary thyroid cancer [7,8,9]. However, many clinical studies revealed unacceptable ocular and neurologic toxicities, and its use as second-line therapy was stopped. We hypothesized that tumor-targeted modification of PD0325901, aiming at improving the tumor uptake and accumulation of this inhibitor, would reduce the undesired systemic toxicity and improve its therapeutic effect.

Peptide-drug conjugation is one of the most promising strategies to overcome the undesired side effects of anti-cancer drugs by the selective delivery of drug conjugates to tumor cells [10]. These peptides recognize and bind to specific receptors with high-affinity, on the tumor-cell surfaces. With subsequent internalization (via receptor-mediated endocytosis) of the ligand-receptor complex, drugs covalently linked to the peptide ligands travel into the targeted tumor cells to exhibit inhibition activity. Among various types of targeting peptides, the ligands containing RGD (Arg-Gly-Asp) amino acid sequences usually have a high affinity for integrin α_v_β_3_ and α_v_β_5_ receptors, membrane proteins abundant on several classes of cancer cells, widely used as carrying vehicles to deliver anti-cancer drugs [11]. In addition, these ligands also worked for integrin targeted delivery of gene therapeutics to tumors with overexpressing integrin receptors [12]. We herein report the conjugation of PD0325901 and its modified analogs with integrin α_v_β_3_ receptor specific peptides c(RGDyk) for the MEK1/2 inhibition in integrin-overexpressing tumor.

**Figure 1:**
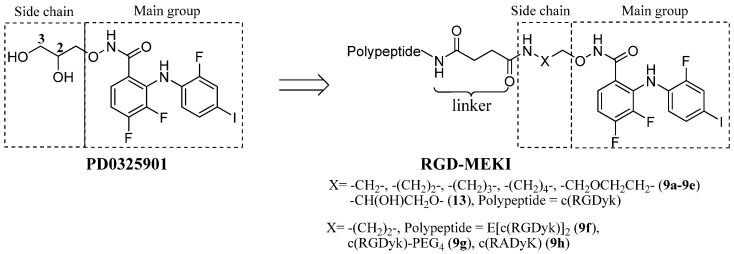
The structures of two series of RGD-PD0325901 conjugates.

Based on the reported X-ray co-crystal structure of a close analogue of PD0325901 with its kinase target, the halo-substituted diphenylamine core and hydroxamate oxygens show important H-bonding interactions with the residues in an allosteric pocket of the target kinase [13], while the dihydroxy propylhydroxamate side chain which showed the improved microsomal stability and solubility of PD0325901, is not directly involved in PD0325901’s binding activity [14,15], and therefore, could be modified by peptide conjugation. In the first series of peptide-drug conjugates, an amine was introduced into PD0325901derivatives (MEKIs, MEK inhibitors) to allow conjugation to the targeting moiety—cRGD peptide via an amide bonds. In order to evaluate the kinase inhibiting and receptor recognizing property of the RGD-MEKI conjugate, a group of MEKIs and RGD-MEKI conjugates with variable chain length were initially evaluated by the introduction of 2–5 carbon linker between the hydroxamate group and the amide group (Figure 1).

We also developed another type of RGD-PD0325901 conjugate with unmodified drug structure and an ester linkage. The hydroxyl group of dihydroxypropyl hydroxamate side chain was carboxylated with succinic anhydride for the tumor targeting peptide cRGD’s conjugation. All the conjugates **9a**–**h** and **13** were investigated for their biological properties, including inhibition to phosphorylated and unphosphorylated MEK1 kinase, ERK pathway inhibition, and anti-proliferation effects.

## 2. Results and Discussion

### 2.1. Development of the RGD-MEKI Conjugates with Alkoxylamine Analog MEKIs

#### 2.1.1. Synthesis of the Alkoxylamine Analog Conjugates **9a–g**

**Scheme 1:**
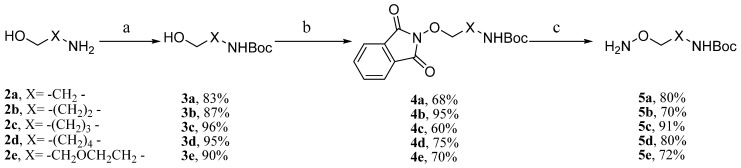
Synthesis of compounds **5a**–**e**.

The synthesis of alkoxylamine analog MEKIs started with preparation of the side chains. As shown in Scheme 1, five side chains **5a**–**e** were synthesized in three steps. After amine protection with (Boc)_2_O in **2a** [16], the hydroxyl group in 2-aminoethanol **3a** was transformed into an 2-alkoxylamine through Mitsunobu reaction and the subsequent hydrazine reduction [17]. Using the same method, the five alkoxylamine analogues 5**a**–**e** were prepared (Scheme 1).

*Reagents and conditions*: (a) (Boc)_2_O, NaOH, dioxane/H_2_O, rt, 12 h; (b) N-hydroxyphthalimide, triphenylphosphine, DEAD, CH_2_Cl_2_, rt, 24 h; (c) NH_2_NH_2_, EtOH, rt, 2 h.

**Scheme 2:**
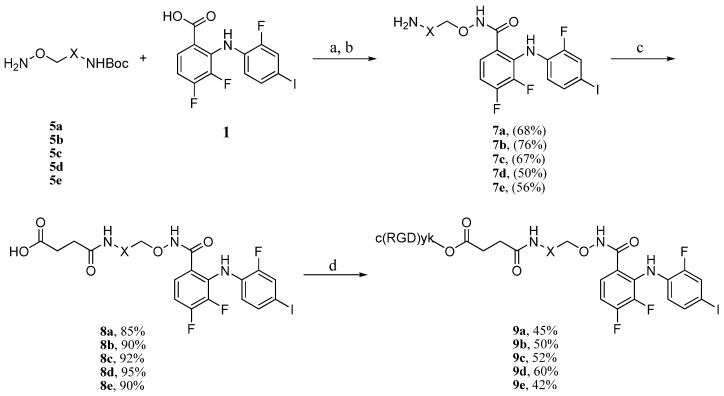
Synthesis of conjugates **9a**–**e**.

According to the previous procedure, coupling of the side chain **5a** with 3,4-difluoro-2-((2-fluoro-4-iodophenyl)amino)benzoic acid (**1**) was then performed. Compound **1** and side chain **5a** were treated with PyBOP in the presence of DIPEA in a mixture of THF and DCM, followed by the removal of the Boc group. PD0325901 analogue **7a** was obtained in 68% yield (two steps) [15,18]. Derivation of the terminal amine group of **7a** with succinic anhydride provided the hemisuccinate ester **8a** in 85% yield [19]. The peptide conjugation reaction was initiated from the activation of **8a** with PyBOP in the presence of DIPEA, followed by the addition of c(RGDyk) peptide, which afforded RGD-MEKI conjugate **9a** in 45% yield. Other peptide conjugates **9b**–**e** were similarly synthesized using the same synthetic route (Scheme 2).

*Reagents and conditions*: (a) DIPEA, PyBOP, THF/CH_2_Cl_2_, rt, 10 h; (b) TFA, CH_2_Cl_2_, rt, 0.5 h; (c) Succinic anhydride, Pyridine, rt, 1 h; (d) iPr_2_NEt, PyBOP, DMF, c(RGDyK), 0 °C, 4 h.

**Scheme 3:**
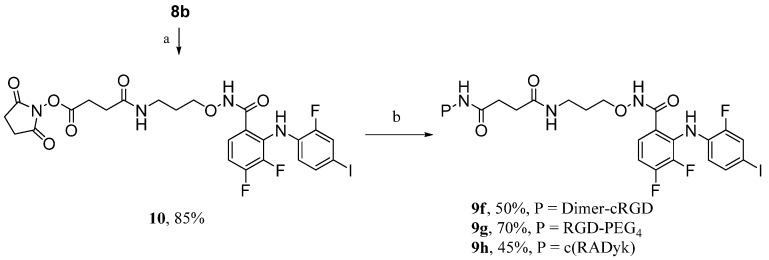
Synthesis of compounds **9f** (dimer-RGD conjugate), **9g** (PEGylated RGD conjugate), and **9h** (scrambled peptide—RAD conjugate).

Based on a receptor-mediated endocytosis mechanism, peptide carriers with higher receptor-binding affinities should greatly promote the inhibitory activity and specificity of RGD-MEKI conjugates for tissue selective targeted cancer treatment. Multimeric cRGD peptides, such as E[c(RGDyK)_2_] (dimer-cRGD) and E{E[c(RGDyK)_2_]_2_} (tetra-cRGD) showed much better tumor targeting capability with higher tumor uptake and longer tumor retention times by compared with their monomeric RGD peptide conjugates [20,21]. Moreover, the PEG linkers are often used in peptide conjugation to increase both the distance and the hydrophilicity of the conjugates. After brief screening of several synthetic methods, a two-step route was finally used for the synthesis of dimer-cRGD and cRGD-PEG_4_ conjugated RGD-MEKI analogs. As shown in Scheme 3, compound **8b** was transformed into the MEKI-succinimydyl ester (MEKI-OSu) **10** in the condition of NHS (*N*-hydroxy succinimide) and DCC in DMF, which then reacted with dimer-cRGD and cRGD-PEG_4_ in base condition to give the corresponding conjugates **9f** and **9g**. In the previous direct activation synthetic method, the dimer-cRGD unit with two free carboxylic acids could be activated by the coupling reagents, leading to a complex reaction mixture. The bio-conjugating method using the activated ester of MEKI **10b** avoided the side reactions and gave dimer-cRGD conjugate **9f** in moderate yield.

*Reagents and conditions*: (a) NHS, DCC, DMF, rt, 24 h; (b) iPr_2_NEt, DMF, dimer-cRGD or cRGD-PEG_4_, rt, 2 h.

#### 2.1.2. Kinase-Inhibiting Activity Study of Conjugates **9a–g**

The kinase-inhibiting activity of RGD-MEKI conjugates **9a**–**g** with alkoxylamine side chains were initially evaluated by Homogenous Time Resolved Fluorescence (HTRF) kinase assays as presented in Table 1. In HTRF phosphor-MEK1 assay, conjugates **9a**–**d** showed higher activity in inhibiting the phosphorylation and activation of inactive MEK1 than in inhibiting the activity of activated MEK1 in the HTRF phosphor-ERK2 assay. The order of inhibition values of **9a**–**d** were **9d** > **9c** > **9b** > **9a **in both assays, indicating that the kinase inhibitory property of the conjugates was only slightly affected by the spacer length between the cRGD vehicle and kinase inhibitor units. Although the activity of these conjugates was not as good as PD0325901 (PD0325901: IC_50_ = 2.6 nM, **9b**: IC_50_ = 47.9 nM, in BRAF-MEK1 assay), they worked as the same non-ATP-competitive inhibition fashion and also similar to the unconjugated compounds. Compared to the mono-cRGD conjugates **9b**, which has the same MEKIs part, the PEGylated conjugate **9g** exhibited increasing activity, and the dimer-cRGD conjugate **9f** showed similar activity as **9b** (Table 1).

**Table 1:** HTRF phosphor-MEK1 assay and HTRF phosphor-ERK2 assay results for PD0325901 and RGD-MEKI conjugates **9a**–**d**, **9f**, **9g** and **13**.

Compound	BRAF-MEK1 assay (%) ^a^			MEK1-ERK2 assay (%) ^b^		A375 cell ^c^
	1 μM	0.1 μM	0.01μM	10 μM	1 μM	IC_50_(μM)
**PD0325901**	97.3 ± 1.9	95.0 ± 1.4	94.0 ± 1.4	100.6 ± 0.4	100.0 ± 1.5	0.00045
**9a**	74.2 ± 7.2	30.0 ± 4.7	0.3 ± 0.0	88.4 ± 1.4	66.5 ± 7.7	4.4
**9b**	77.6 ± 7.5	58.5 ± 8.5	6.7 ± 8.5	93.5 ± 2.2	69.9 ± 3.2	2.1
**9c**	91.5 ± 0.7	48.5 ± 9.5	38.9 ± 10	99.5 ± 0.7	63.8 ± 2.9	4.0
**9d**	97.9 ± 2.4	46.0 ± 12.5	26.1 ± 15	99.9 ± 1.4	80.5 ± 2.0	5.6
**8b**	57.5 ± 1.7	39.6 ± 9.3	12.5 ± 3.2	83.4 ± 1.2	29.1 ± 12.0	14.2
**9g**	78.8 ± 4.4	39.0 ± 7.2	25.9 ± 11	91.8 ± 2.5	40.6 ± 5.5	0.65
**9f**	80.5 ± 2.9	42.6 ± 5.6	39.0 ± 7.4	95.0 ± 1.0	49.1 ± 7.8	3.1
**13**	95.7 ± 1.9	95.1 ± 0.9	48.4 ± 3.2	-	-	0.0176

^a^ BRAF-MEK1 assay (HTRF phosphor-MEK1 assay) was used to determine the activity of RGD-MEKI conjugates to inhibit the phosphorylation of the inactive MEK1 kinase. Values are means of three experiments, standard deviation is given after them; ^b^ MEK1-ERK2 assay (HTRF phosphor-ERK2 assay) was used to determine the activity of RGD-MEKI conjugates to inhibit the active MEK1 kinase to phosphorylate the inactive ERK2 protein. Values are means of three experiments; standard deviation is given after them; ^c^ *In vitro* anti-proliferation assay on melanoma A375 cells by the SRB method.

#### 2.1.3. *In Vitro* Anti-Proliferation Assay of Conjugates **9a–g**

*In vitro* properties of RGD-MEKI conjugates **9a**–**d** were evaluated against melanoma A375 cells by the SRB method. A375 cells which harbor activating BRAF^V600E^ mutation (the valine 600 to glutamate mutation of BRAF kinase) with higher ERK pathway activity are exquisitely sensitive to PD0325901 treatment. All the conjugates exhibited moderate cytotoxicity in A375 cell-proliferation assay in which **9b** exhibited a better inhibition activity (Table 1).Although increasing the space length between the modified PD0325901 and peptide cargo is beneficial to the kinase activity of alkoxylamine analog conjugates, we considered that a longer carbon chain would decrease the aqueous solubility of conjugates, and thus influence the conjugate’s cell-based activity. In an effort to combine long chain length with better hydrophilicity, the RGD-MEKI conjugate **9e** was designed by using an ethoxyethyl amine unit instead of the alkoxylamine structure. However, **9e** showed much lower activity than its carbon chain homologues **9d** in cytotoxicity assay.

**Figure 2:**
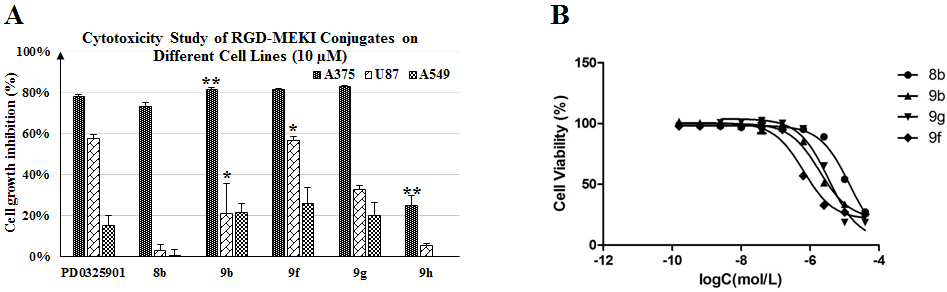
(**A**) Cytotoxicity study of RGD-MEKI conjugates **9b**, **9f**–**h**, compared with the parent drug (PD0325901) and MEKI—**8b** on different tumor cell lines (melanoma-A375, glioblastoma-U87, NSCLC-A549) at 10 µM concentration by the SRB method. (**B**) IC_50_ value of **8b**, **9b**, **9f** and **9g **in A375 cells. The effect of cRGD peptide cargo on conjugates was analyzed by student’s *t*-test, and (*) indicates *p* value < 0.05, (**) indicates *p* value < 0.01.

The cytotoxic potency and selectivity of conjugates **9b**, **9f** and **9g** were measured against three different tumor cell lines, including A375, U87 (glioblastoma) and A549 (NSCLC). Different from melanoma A375, U87 cells were found to be moderately sensitive to PD0325901, while A549 was the lowest. As shown in Figure 2, **9f** and **9g** exhibited selective cytotoxicity against the tested carcinoma cell lines (A375 > U87 > A549) as the same order observed in PD0325901. As shown in Table 1 and Figure 2B, the hydrophilic PEG_4_ linker improved the cytotoxic potency of **9g **with a sub-µM IC_50_ value (0.65 ± 0.03 µM) in A375 proliferation assay, which is 3-fold more potent than its homolog **9b **(2.1 ± 0.8 µM). This indicated that longer and hydrophilic spacer between MEKI and peptide cargo was beneficial for the ligand-to-receptor-binding and inhibitor-kinase-interaction. However, in the less sensitive A549 proliferation assay **9b** and **9g** were found to exhibit similar lower growth-inhibitory activity as PD0325901.

Although the dimer-cRGD conjugate **9f** (IC_50_ = 3.0 ± 1.3 µM) was found to be less potent than the monomer conjugate analog **9b** (IC_50_ = 2.1 ± 0.8 µM) in A375 proliferation assay, it showed the best U87 cell growth-inhibiting activity in all alkoxylamine analogs RGD-MEKIs, much better than mono- RGD conjugate (* *p* < 0.05). Especially, its activity was as good as that of PD0325901 at a high concentration (10 µM). U-87 cells, human neuronal glioblastoma cells, expressed a high level of integrin α_v_β_3_ receptors on cell surface with a ratio of receptors/cell (cell receptor density) up to (1.28 ± 0.46) × 10^5^ [22]. We considered that the better receptor-binding affinity exhibited by the dimer-cRGD conjugate **9f** could enhance its antitumor activity against the receptor overexpressed U87 cells. This specific receptor-mediated tumor inhibition activity could be blocked by the scramble peptide—c(RADyK) conjugated MEKI—**9h** (Scheme 3). As shown in Figure 2, the A375 cell-growth inhibition was decreased from 81.4% (**9b**) to 25.1% (**9h**) (** *p* < 0.01), and the U87 cell-growth inhibition was decreased from 21.3% (**9b**) to 5.4% (**9h**), respectively.

#### 2.1.4. Inhibition of ERK Pathway

**Figure 3:**
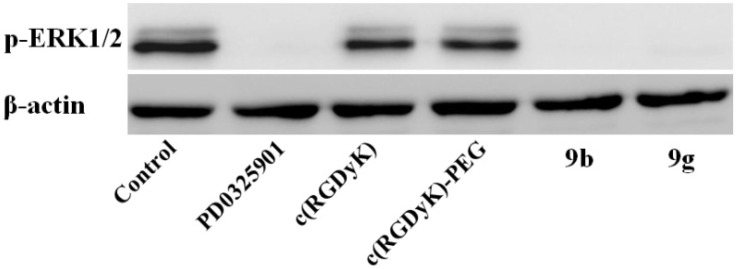
Western blotting analyses of melanoma A375 cells lysates were performed for p-ERK1/2 after 2h. Β-actin served as loading control. Cells were treated with 10 μM of **PD0325901**, **c(RGDyK)** peptides, and RGD-MEKI conjugates **9b** and **9g**.

Inhibition of the ERK phosphorylation has been proposed as a primary biomarker of MEK inhibition activity [23,24]. As exhibited in Figure 3A, western blotting analyses showed that up to 90% inhibition of the phosphorylation of ERK1/2 protein (44/42 kDa) was observed in conjugate **9b** and **9g**—treated A375 cells, which is the same as the parent drug, while the cRGD peptides themselves exhibited no inhibitory activity (Figure 3).

#### 2.1.5. The Cell Cycle Analysis of Conjugates

**Figure 4:**
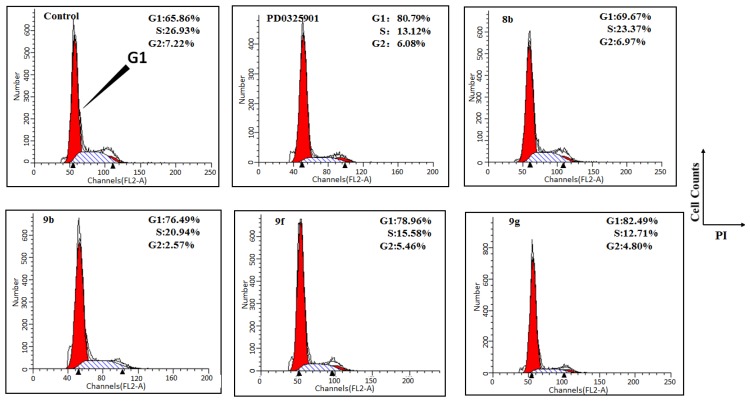
RGD-MEKI induced cell cycle arrest in melanoma A375 cells.

Although these newly developed alkoxylamine analogs MEKIs conjugates did not show attractive tumor-inhibition properties, multivalent RGD ligands enhanced receptor-mediated drug endocytosis in receptor over-expressed tumor cells, as evidenced by **9f**, and PEGylation improved the cell-based anti-proliferation properties of conjugate **9g**. Cell-cycle analysis revealed that A375 cell-growth reduction was associated with loss of cells in S and G2 phase (Figure 4). Cell proliferation is regulated during the first pause in the cell cycle at the G1 checkpoint.

Cells were treated with 10 µM of PD0325901 and RGD-MEKI for 24 h, stained with PI and subjected to FACS analysis.

We tried to improve the conjugate’s hydrophilicity in **9e** and **9f**, but **9e** showed a decreased activity compared to its chain analog **9d**. We suggest that **9e**’s poor performance might be attributed to the unfavored side-chain modification. As compared with the side-chain structure of the lead compound PD0325901, the hydroxyl methylene group in PD0325901 was replaced by an oxygen atom in conjugate **9e** (IC_50_ = 9.5 ± 0.16 µM), in which, the former unit can act as the hydrogen-bond donor, while the latter one can only act as the hydrogen-bond acceptor. The relative lower biological activity of conjugate **9e** might be due to the modified electron density, or more specific to the inverted hydrogen bonding potential. Thus, we suggest that C2-hydroxy group of dihydroxypropyl side chain might be essential to PD0325901’s biological activity, not only by increasing its aqueous solubility. Further exploration of structural requirements led to the synthesis of the RGD-PD0325901 direct conjugate **13**, in which, the terminal hydroxyl group of PD0325901 was carboxylated with succinic anhydride for the tumor-targeting peptide cRGD’s conjugation.

### 2.2. Development of the RGD-PD0325901 Direct Conjugates **13**

#### 2.2.1. Synthesis of Conjugate **13**

**Scheme 4:**
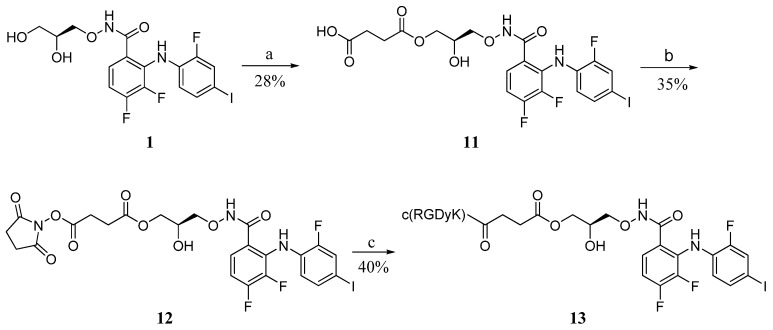
Synthesis of the RGD-PD0325901 direct conjugate **13**.

Following the previous procedure, PD0325901 **1** was transformed into the hemisuccinate ester **11** by reacting with succinic anhydride and pyridine. Subsequent activation of the free carboxylic acid with NHS (*N*-hydroxy succinimide) and DCC in DMF, followed by the reaction with c(RGDyk) gave the corresponding conjugates **13** in a moderate yield (Scheme 4).

*Reagents and conditions*: (**a**) Succinic anhydride, pyridine, rt, 1 h; (**b**) NHS, DCC, DMF, rt, 24 h; (**c**) iPr_2_NEt, DMF, rt, 2 h.

#### 2.2.2. *In Vitro* Biological Evaluation of Conjugate **13**

**Figure 5:**
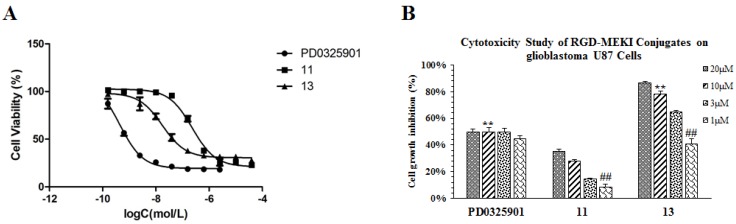
Cytotoxicity study of conjugate **13**, compared with parent drug (PD0325901) and MEKI (**11**) on both melanoma A375 (**A**, IC_50_ value) and glioblastoma-U87 (**B**) cells after 72h treatment by the SRB method.

To our delight, this new RGD-MEKI conjugate **13** showed potent enzymatic activity in phosphor-MEK1 assay (Table 1), and also displayed comparable cellular activity (IC_50_ = 17.6 nM) to its parent drug—PD0325901 (IC_50_ = 0.47 nM) in the A375 proliferation assay (Figure 5A). Without the cRGD cargo, its analogue **11** lost almost 10-fold antitumor efficacy (IC_50_ value of 230 nM) compared to **13**, Furthermore, conjugate **13** exhibited higher anti-proliferative activity than PD0325901 in U87 glioblastoma cells with an IC_50_ value of 2.38 µM, while PD0325901 showed around 50% inhibitory activity at high concentrations (>1 µM) in a concentration-independent manner (Figure 5B). All these results demonstrated that the RGD-PD0325901 conjugate **13 **enters tumor cells through a receptor-mediated mechanism in integrin α_v_β_3_-overexpressing cells.

(**) indicates significant deference between conjugate **13** and parent drug PD0325901 (*p* value < 0.01). (##) indicates *p* value < 0.01.

**Figure 6:**
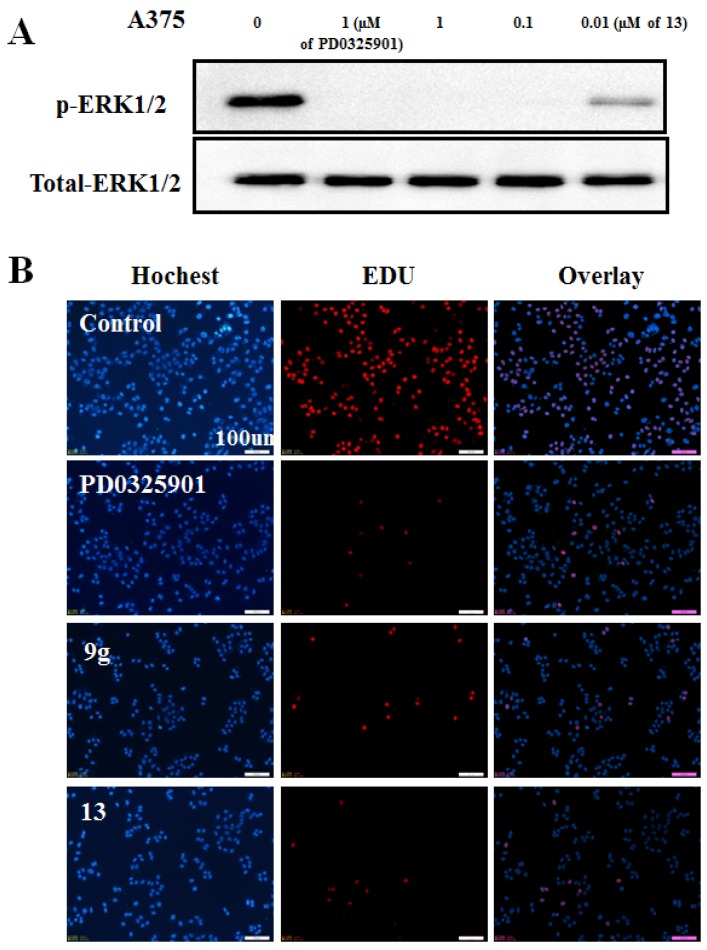
(**A**) Western blots analyses of melanoma A375 cells lysates were performed for p-ERK1/2 after 2 h*. (**B**) DNA replication study of **9f** and **13** on A375 cells by EdU method *.

Consistent with its anti-proliferation effects, the conjugate **13** showed a dose-dependent pERK1/2 suppression in A375 cells (Figure 6A), with up to 90% inhibition of pERK1/2 protein at 0.01 µM concentration. This result was in agreement with the inhibition manner of DNA replication. As shown in Figure 6B, conjugate **13** and **9g** inhibited DNA replication of A375 cells with the same efficiency as the parent drug PD0325901.

*A Total ERK1/2 served as loading control. Cells were treated with 1 uM of **PD0325901** and different concentrations of conjugate **13**. *B EdU (5-ethynyl-2′-deoxyuridine) was stained cells (red) after 24 h drug treatment. The nuclei of total cells were stained with Hochest33342 (blue).

In order to increase the therapeutic efficacy of PD0325901 while decreasing it systemic toxicity, the cleavable carboxylic acid ester bond was introduced into the RGD-MEKI conjugates. In our study, this conjugate **13** exhibited specific and potent activity in MEK enzyme inhibition, anti-cell-proliferation, p-ERK-1/2 inhibition, and DNA replication inhibition. All these data demonstrate that conjugate **13** was potential MEK1/2 kinase inhibitor with improved tumor targeting capability. The ester bond of molecules can be hydrolyzed by either blood or cellular esterases. How stable the conjugate is in blood circulation system and if it can be cleaved to release drug in target cancer cells are two important properties of prodrugs. In the further research, we will focus on the study to answer these two questions as well as the pharmacological activity study of RGD-MEKI conjugate.

## 3. Experimental

### 3.1. General Experimental Conditions

All reactions were run under an inert atmosphere (N_2_) with flame-dried glassware using standard techniques for manipulating air-sensitive compounds. Commercial reagents were used as supplied or purified by standard techniques where necessary. THF and CH_2_Cl_2_ were used fresh distilled over sodium/benzophenone or calcium hydride respectively. The cyclic RGD peptides, such as c(RGDyk), c(RGDyk)-PEG_4_ (cRGD-PEG_4_), c(RADyk), and E[c(RGDyK)_2_] (dimer-cRGD) were purchased from GL Biochem (Shanghai) Ltd., with the purity higher than 95%. Column chromatography was performed using 200-300 mesh silica with the proper solvent system according to TLC analysis using KMnO_4_ stain and UV light to visualize the reaction components. Unless otherwise noted, ^1^H-NMR (400 MHz), ^19^F-NMR spectra (376 MHz) were recorded at ambient temperature using a BrukerAvance III400 MHz spectrometer (Bruker, Karlsruhecity, Germany). NMR data were reported as follows: chemical shift, multiplicity (s = singlet, d = doublet, t = triplet, m = multiplet and bs = broad singlet), coupling constant in Hz and integration. Chemical shifts are expressed in ppm relative to tetramethylsilane. IR spectra were recorded on a FTIR spectrometer (IR Prestige-21, Shimadzu, Kyoto, Japan) in KBr pellets and reported in reciprocal centimeters (cm^−1^). Low-resolution MS and HRMS data were obtained using ESI ionization (Bruker DALTONICS APEX IV 70e, Germeringcity, Germany). All melting points were taken with an X-6melting point apparatus (Beijing Focus Instrument Co., Ltd., Beijing, China) and are reported without correction. Analytical as well as semi-preparative reversed-phase high-performance liquid chromatography (RP-HPLC) were performed on a LC3000 Binary chromatography system with a UV3000 UV-VIS Detector (CXTH, Beijing Chuangxintongheng Science & Technology Co., Ltd., Beijing, China).The semi-preparative HPLC was performed with a Daisogel C18 10 μm 100A column (10 micron, 250 × 30 mm). The flow was 20 mL/min, with the mobile phase starting from 95% solvent A (0.1% TFA in water) and 5% solvent B (0.1% TFA in acetonitrile) (0–5 min) to 5% solvent A and 95% solvent B at 45 min. Analytical HPLC was performed using the same gradient system, but with a Gemini 5 μm C18 110A column (250 × 10 mm) and flow of 2 mL/min. Ultraviolet (UV) absorbance was monitored at 254 nm.

### 3.2. Chemistry

#### 3.2.1. General Procedure for the Synthesis of Tert-butyl-(2-hydroxyethyl)carbamate **3a** and Its Derivatives (**3b–e**) [25,26,27,28,29]

To a solution of **2a** (or **2b**–**e**, 20 mmol) in a mixture of dioxane (15 mL) and H_2_O (7 mL), was added 5N NaOH (4.8 mL) and a solution of Boc_2_O (5.0 g, 23 mmol) in dioxane at 0 °C. After stirred at rt overnight, the reaction mixture was concentrated in vacuo. The residue was extracted from 10% citric acid with AcOEt, and dried over anhydrous Na_2_SO_4_. Evaporation of the solvents gave the pure product **3a**–**e**. **3a** was obtained as a colorless oil (2.67 g, 83%).

#### 3.2.2. General Procedure for the Synthesis of Tert-butyl-(2-((1,3-dioxoisoindolin-2-yl)oxy)ethyl) carbamate **4a** and Its Derivatives (**4b–e**) [30,31,32,33]

To a solution of **3a** (or **3b**–**e**, 10 mmol), *N*-hydroxy-phthalimide (1.79 g, 11 mmol), and triphenylphosphine (2.88 g, 11 mmol) in CH_2_Cl_2_ (25 mL), was added diethyl azodicarboxylate (DEAD, 1.92 g, 11 mmol) at 0 °C. The reaction mixture was stirred at rt overnight. The reaction was concentrated and purified by column chromatography to give the product **4a**–**e**. **4a** was obtained as a white solid (2.08 g, 68%).

#### 3.2.3. General Procedure for the Synthesis of Tert-butyl-(2-(aminooxy)ethyl) carbamate **5a** and Its Derivatives (**5b–e**) [32,33,34]

To a solution of **4a** (or **4b**–**e**, 5.8 mmol ) in EtOH (10 mL), was added hydrazine (187 uL, 5.97 mmol) in drop. The reaction mixture was stirred at rt. until the reaction was completed as judged by TLC. The mixture was filtered and the filtrate was concentrated and purified by column chromatography to give the product **5a**–**e**. **5a** was obtained as a white solid (817 mg, 80%).

#### 3.2.4. General Procedure for the Synthesis of Tert-butyl-(2-((3,4-difluoro-2-(2-fluoro-4-iodobenzyl) benzamido)oxy)ethyl)carbamate **6a** and Its Derivatives (**6b–e**)

To a stirring mixture comprised of 3,4-difluoro-2-((2-fluoro-4-iodophenyl)amino)benzoic acid (755 mg, 1.92 mmol), **5a** (or **5b**–**e**, 2 mmol) and diisopropylethylamine (0.7 mL, 4.23 mmol) in 40 mL of 1:1 (v/v) THF-DCM, was added directly the benzotriazole-1-yl-oxy-*tris*-pyrrolidino-phosphonium hexafluorophosphate (PyBOP) (1.04 g, 2 mmol). The reaction mixture was stirred at room temperature under argon atmosphere overnight. The mixture was concentrated to an oil under reduced pressure. The oil was partitioned between diethyl ether (60 mL) and 10% aqueous hydrochloric acid (45 mL). The organic phase was washed with saturated aqueous sodium bicarbonate (40 mL) and brine (45 mL), and then dried with MgSO_4_, concentrated in vacuo. The residue was purified through column chromatography to give the product **6a**–**e**.

*Tert-butyl-(2-((3,4-difluoro-2-(2-fluoro-4-iodobenzyl)benzamido)oxy)ethyl)carbamate* (**6a**). **6a** was obtained as a brown solid (592 mg, 83%). Mp. 61.2–63.2 °C; IR (neat) 1666, 1602, 1494, 1288, 1166; ^1^H NMR (400 MHz, CDCl_3_) *δ* 10.44 (br, 1H), 8.58 (br, 1H), 7.37(dd, *J* = 10.2, 1.4 Hz, 1H), 7.34 (t, *J* = 7.3 Hz, 1H), 7.30 (d, *J* = 8.5 Hz, 1H), 6.87–6.81 (m, 1H), 6.59–6.54 (m, 1H), 5.18 (br, 1H), 3.98–3.85 (m, 2H), 3.40–3.36 (m, 2H), 1.43 (s, 9H); ^19^F NMR (376 MHz, CDCl_3_) *δ* −127.78, −129.53, −142.35; HRMS (ESI) calcd. for C_20_H_22_F_3_IN_3_O_4_ [M+H]^+^ 552. 0607; found, 552.0601.

*Tert-butyl(3-((3,4-difluoro-2-((2-fluoro-4-iodophenyl)amino)benzamido)oxy)propyl)carbamate* (**6b**). **6b** was obtained as a white solid (82% yield); mp. 138.5–139.5 °C; IR (neat) 1681, 1602, 1496, 1276, 1168; ^1^H NMR (400 MHz, CDCl_3_) *δ* 9.97 (br, 1H), 8.60 (br, 1H), 7.40–7.37 (m, 2H), 7.31 (d, *J* = 8.5 Hz, 1H), 6.83–6.77 (m, 1H), 6.60–6.55 (m, 1H), 5.10 (br, 1H), 4.02 (t, *J* = 5.6 Hz, 2H), 3.33–3.30 (m, 2H), 1.84–1.78 (m, 2H), 1.42 (s, 9H); ^19^F NMR (376 MHz, CDCl_3_) *δ* −127.99, −129.13, −142.75(d, *J* = 19.0Hz); HRMS (ESI) calcd for C_21_H_24_F_3_IN_3_O_4_ [M+H]^+^ 566.0764; found, 566.0745.

*Tert-butyl(4-((3,4-difluoro-2-((2-fluoro-4-iodophenyl)amino)benzamido)oxy)butyl)carbamate* (**6c**). **6c** was obtained as a brown solid; yield 70%; mp. 125.1–127.1 °C; IR (neat) 1683, 1602, 1498, 1276, 1170; ^1^H NMR (400 MHz, CDCl_3_) *δ* 10.74 (br, 1H), 8.57 (br, 1H), 7.35 (t, J = 7.3 Hz, 1H), 7.34 (dd, *J* = 10.2, 1.4 Hz, 1H), 7.26 (d, *J* = 7.4 Hz, 1H), 6.77–6.71 (m, 1H), 6.55–6.50 (m, 1H), 4.84 (br, 1H), 3.94–3.92 (m, 2H), 3.15–3.13 (m, 2H), 1.61–1.54 (m, 4H), 1.37 (s, 9H); ^19^F NMR (376 MHz, CDCl_3_) *δ *−127.59, −130.39, −142.35; HRMS (ESI) calcd. for C_22_H_26_F_3_IN_3_O_4_ [M+H]^+^ 580.0920; found, 580.0892.

*Tert-butyl(5-((3,4-difluoro-2-((2-fluoro-4- iodophenyl)amino)benzamido)oxy)pentyl)carbamate* (**6d**)*.* **6d** was obtained as a white solid (70% yield); mp. 133.1–135.1 °C; IR (neat) 1681, 1602, 1496, 1276, 1168; ^1^H NMR (400 MHz, CDCl_3_) *δ* 10.17 (br, 1H), 8.06 (br, 1H), 7.44 (t, *J* = 6.9 Hz, 1H), 7.39 (dd, *J* = 10.3, 1.8 Hz, 1H), 7.30 (d, *J* = 8.5 Hz, 1H), 6.90–6.84 (m, 1H), 6.58–6.52 (m, 1H), 3.92 (t, *J* = 5.8 Hz, 2H), 3.14 (t, *J* = 5.8 Hz, 2H), 1.69–1.63 (m, 2H), 1.53–1.46 (m, 4H), 1.41 (s, 9H); ^19^F NMR (376 MHz, DMSO) *δ* −127.82, −130.17, −142.63; HRMS (ESI) calcd. for C_23_H_28_F_3_IN_3_O_4_ [M+H]^+^ 594.1077; found, 594.1037.

*Tert-butyl(2-(2-((3,4-difluoro-2-((2-fluoro-4-iodophenyl)amino)benzamido)oxy)ethoxy)ethyl) carbamate* (**6e**)*.* **6e** was obtained as a brown solid (74% yield); mp. 78.5–79.5 °C; IR (neat) 1681, 1602, 1496, 1278, 1068; ^1^H NMR (400 MHz, CDCl_3_) *δ* 10.16 (br, 1H), 8.52 (br, 1H), 7.45 (t, *J* = 5.6 Hz, 1H), 7.38 (dd, *J* = 10.3, 1.8 Hz, 1H), 7.29 (d, *J* = 8.5 Hz, 1H), 6.87–6.81 (m, 1H), 6.58–6.52 (m, 1H), 4.11 (t, *J* = 3.9 Hz, 2H), 3.71 (t, *J* = 4.2 Hz, 2H), 3.54 (t, *J* = 4.8 Hz, 2H), 3.33 (t, *J* = 4.8 Hz, 2H), 1.38 (s, 9H); ^19^F NMR (376 MHz, CDCl_3_) *δ* −127.57, −130.74, −142.26; HRMS (ESI) calcd. For C_22_H_26_F_3_IN_3_O_5_ [M+H]^+^ 596.0869; found, 596.0827.

#### 3.2.5. General Procedure for the Synthesis of N-(2-aminoethoxy)-3,4-difluoro-2-((2-fluoro-4-iodophenyl)amino)benzamide **7a** and Its Derivatives (**7b−e**)

To a solution of **6a** (or **6b**−**e**, 2.36 mmol) in CH_2_Cl_2_ (5 mL) was added trifluoroacetic acid (5 mL, 50 v/v %), and the mixture was stirred at 0 °C for 1 h. The mixture was diluted with AcOEt and was then basified to pH > 7.0 by the addition of aqueous NaOH. The organic phase was then sequencely washed with saturated aqueous sodium bicarbonate and brine, was dried (MgSO_4_), and was concentrated and purified by column chromatography to give the product **7a**−**e**.

*N-(2-aminoethoxy)-3,4-difluoro-2-((2-fluoro-4-iodophenyl)amino)benzamide* (**7a**)*.* **7a** was obtained as a white solid (1.0 g, 95%); mp. 186.5−187.5 °C; IR (neat) 1683, 1496, 1288, 1136, 1047; ^1^H NMR (400 MHz, DMSO) *δ* 7.67 (t, *J* = 7.2 Hz, 1H), 7.57 (dd, *J* = 10.8, 1.7 Hz, 1H), 7.37 (d, *J* = 8.5 Hz, 1H), 7.08–7.01 (m, 1H), 6.63−6.57 (m, 1H), 3.98−3.85 (m, 2H), 2.94−2.90(m, 2H); ^19^F NMR (376 MHz, DMSO) *δ *−130.81, −136.27, −146.62 (d, *J* = 20.1Hz); HRMS (ESI) calcd. for C_15_H_14_F_3_IN_3_O_2_ [M+H]^+^ 452.0083; found, 452.0065.

*N-(3-aminopropoxy)-3,4-difluoro-2-((2-fluoro-4-iodophenyl)amino)benzamide* (**7b**)*.* **7b** was gave as a white solid (90% yield); mp. 197.5−198.5 °C; IR (neat) 1602, 1496, 1327, 1122, 1045; ^1^H NMR (400 MHz, DMSO) *δ* 7.69 (t, *J* = 7.0 Hz, 1H), 7.56 (dd, *J* = 10.7, 1.8 Hz, 1H), 7.36 (d, *J* = 8.4 Hz, 1H), 7.04−6.97 (m, 1H), 6.62−6.56 (m, 1H), 3.83−3.81 (m, 2H), 2.92–2.89 (m, 2H), 1.79−1.76 (m, 2H); ^19^F NMR (376 MHz, MeOD) *δ *−128.52, −132.20 (d, *J* = 17.8Hz), −144.53 (d, *J* = 18.5Hz); HRMS (ESI) calcd. for C_16_H_16_F_3_IN_3_O_2_ [M+H]^+^ 466.0239; found, 466.0225.

*N-(4-aminobutoxy)-3,4-difluoro-2-((2-fluoro-4-iodophenyl)amino)benzamide* (**7c**)*.* **7c** was obtained as a brown solid (95% yield); mp. 146.7–147.7 °C; IR (neat) 1602, 1496, 1290, 1138, 1045; ^1^H NMR (400 MHz, DMSO) *δ* 7.57 (dd, *J* = 10.7, 1.5 Hz, 1H), 7.45 (t, *J* = 7.0 Hz, 1H), 7.37 (d, *J* = 8.5 Hz, 1H), 7.21−7.15 (m, 1H), 6.69−6.63 (m, 1H), 3.83−3.80 (m, 2H), 2.85−2.82 (m, 2H), 1.70−1.62 (m, 4H); ^19^F NMR (376 MHz, DMSO) *δ* −131.47, −140.30, −146.52 (d, *J* = 12.1Hz); HRMS (ESI) calcd. For C_17_H_18_F_3_IN_3_O_2_ [M+H]^+^ 480.0396; found, 480.0372.

*N-((5-aminopentyl)oxy)-3,4-difluoro-2-((2-fluoro-4-iodophenyl)amino)benzamide* (**7d**). **7d** was obtained as a white solid (72% yield); mp. 88.5−89.5 °C; IR (neat) 1678, 1496, 1276, 1203, 1060; ^1^H NMR (400 MHz, DMSO) *δ* 11.81 (br, 1H), 8.71 (br, 1H), 7.73 (br, 1H), 7.59 (dd, *J* = 10.8, 1.7 Hz, 1H), 7.41−7.36 (m, 2H), 7.25−7.19 (m, 1H), 6.70−6.64 (m, 1H), 3.78 (t, *J* = 5.9 Hz, 2H), 2.80–2.75 (m, 2H), 1.56−1.53 (m, 4H), 1.42−1.37 (m, 2H); ^19^F NMR (376 MHz, DMSO) *δ* −129.61, −134.57(d, *J* = 19.7 Hz), −145.69(d, *J* = 19.2Hz); HRMS (ESI) calcd. for C_18_H_20_F_3_IN_3_O_2_ [M+H]^+^ 494.0552; found, 494.0540.

*N-(2-(2-aminoethoxy)ethoxy)-3,4- difluoro-2-((2-fluoro-4-iodophenyl)amino)benzamide* (**7e**)*.* **7e** was obtained as a white solid (75% yield); mp. 197.5–198.5 °C; IR (neat) 1598, 1494, 1354, 1288, 1124; ^1^H NMR (400 MHz, DMSO) *δ* 7.58 (dd, *J* = 10.8, 1.3 Hz, 1H), 7.48 (t, *J* = 6.1 Hz, 1H), 7.37(d, *J* = 8.4 Hz, 1H), 7.21−7.15 (m, 1H), 6.68−6.62 (m, 1H), 3.95 (t, *J* = 4.6 Hz, 2H), 3.64−3.61 (m, 4H), 2.97 (t, *J* = 4.8 Hz, 2H); ^19^F NMR (376 MHz, DMSO) *δ *−130.92, −136.75, −146.75 (d, *J* = 20.5Hz); HRMS (ESI) calcd. for C_17_H_18_F_3_IN_3_O_3_ [M+H]^+^ 496.0345; found, 496.0318.

#### 3.2.6. General Procedure for the Synthesis of 4-((2-((3,4-Difluoro-2-((2-fluoro-4-iodophenyl)amino) benzamido)oxy)ethyl)amino)-4-oxobutanoic Acid **8a** and Its Derivatives (**8b−e**)

A solution of **7a** (or **7b**−**e**, 0.36 mmol) and succinic anhydride (470 mg, 4.6 mmol) in pyridine (7 mL) was stirred for 30 min. The mixture was diluted with AcOEt and then washed with 1M HCl. The organic phase was dried (MgSO_4_), and was concentrated and purified by column chromatography to give the product **8a**−**e**.

*4-((2-((3,4-Difluoro-2-((2-fluoro-4-iodophenyl)amino)benzamido)oxy)ethyl)amino)-4-oxobutanoic acid* (**8a**). **8a** was obtained as a white solid (168 mg, 85%); mp. 96.5−98.5 °C; IR (neat) 1714, 1631, 1604, 1496, 1278; ^1^H NMR (400 MHz, CDCl_3_) *δ* 10.51 (br, 1H), 8.40 (br, 1H), 7.38 (t, *J* = 7.3 Hz, 1H), 7.35(dd, *J* = 10.2, 1.4 Hz, 1H), 7.29(d, *J* = 8.5 Hz, 1H), 6.86−6.80(m, 1H), 6.56−6.51 (m, 1H), 3.92−3.88 (m, 2H), 3.47−3.43 (m, 2H), 2.66−2.62 (m, 2H), 2.56−2.52 (m, 2H); ^19^F NMR (376 MHz, DMSO) *δ* −129.02, −133.97, −145.13(d, *J* = 18.3 Hz); HRMS (ESI) calcd. for C_19_H_18_F_3_IN_3_O_5_ [M+H]^+^ 552.0243; found, 552.0203.

*4-((3-((3,4-Difluoro-2-((2-fluoro-4-iodophenyl)amino)benzamido)oxy)propyl)amino)-4-oxobutanoic acid* (**8b**)*.* **8b** was obtained as a white solid (90% yield); mp. 161.5−162.5 °C; IR (neat) 1726, 1645, 1604, 1494, 1280; ^1^H NMR (400 MHz, CDCl_3_) *δ* 10.55 (br, 1H), 8.37 (br, 1H), 7.90 (br, 1H), 7.40 (t, *J* = 6.7 Hz, 1H), 7.36 (dd, *J* = 10.3, 1.8 Hz, 1H), 7.28 (d, *J* = 8.5 Hz, 1H), 6.88–6.81 (m, 1H), 6.55−6.49 (m, 1H), 3.97−3.95 (m, 2H), 3.40−3.36 (m, 2H), 2.61–2.53 (m, 4H), 1.76−1.73 (m, 2H); ^19^F NMR (376 MHz, DMSO) *δ *−129.05, −134.06 (d, *J* = 18.1 Hz), −145.20 (d, *J* = 19.1Hz); HRMS (ESI) calcd. for C_20_H_20_F_3_IN_3_O_5_ [M+H]^+^ 566.0399; found, 566.0377.

*4-((4-((3,4-Difluoro-2-((2-fluoro-4-iodophenyl)amino)benzamido)oxy)butyl)amino)-4-oxobutanoic acid* (**8c**). **8c** was obtained as a white solid (92% yield); mp. 120.9−121.9 °C; IR (neat) 1716, 1647, 1606, 1496, 1278; ^1^H NMR (400 MHz, DMSO) *δ* 11.67 (br, 1H), 8.71 (br, 1H), 7.83 (br, 1H), 7.57 (dd, *J* = 10.8, 1.4 Hz, 1H), 7.38 (t, *J* = 5.6 Hz, 1H),7.36 (d, *J* = 7.5 Hz, 1H), 7.23−7.16 (m, 1H), 6.70−6.65 (m, 1H), 3.80−3.77 (m, 2H), 3.07−3.03 (m, 2H), 2.41−2.40 (m, 2H), 2.31-2.28(m, 2H), 1.55−1.47 (m, 4H); ^19^F NMR (376 MHz, DMSO) *δ *−129.04, −134.14 (d, *J* = 18.6 Hz), −145.25 (d, *J* = 19.2Hz); HRMS (ESI) calcd. for C_21_H_22_F_3_IN_3_O_5_ [M+H]^+^ 580.0556; found, 580.0530.

*4-((5-((3,4-Difluoro-2-((2-fluoro-4-iodophenyl)amino)benzamido)oxy)pentyl)amino)-4-oxobutanoic acid* (**8d**). **8d** was obtainedas a white solid (95% yield); mp. 186.5−187.5 °C; IR (neat) 1714, 1651, 1608, 1496, 1278; ^1^H NMR (400 MHz, DMSO) *δ* 11.76(br, 1H), 8.70(br, 1H), 7.82(t, *J* = 5.4 Hz, 1H), 7.58 (dd, *J* = 10.8, 1.6 Hz, 1H), 7.40−7.35(m, 2H), 7.24−7.18(m, 1H), 6.69-6.64 (m, 1H), 3.76 (t, *J* = 6.1 Hz, 2H), 3.01 (q, *J* = 6.0 Hz, 2H), 2.41 (t, *J* = 6.6 Hz, 2H), 2.29 (t, *J* = 6.6 Hz, 2H), 1.57−1.50 (m, 2H), 1.40−1.31 (m, 4H); ^19^F NMR (376 MHz, DMSO) *δ *−129.07, −134.21 (d, *J* = 19.3Hz), −145.349 (d, *J* = 19.3Hz); HRMS (ESI) calcd. for C_22_H_24_F_3_IN_3_O_5_ [M+H]^+^ 594.0712; found, 594.0695.

*1-(3,4-Difluoro-2-((2-fluoro-4-iodophenyl)amino)phenyl)-1,10-dioxo-3,6-dioxa-2,9-diazatridecan-13-oic acid* (**8e**). **8e** was obtainedas a white solid (90% yield); mp. 135.1–136.1 °C; IR (neat) 1714, 1651, 1608, 1496, 1280; ^1^H NMR (400 MHz, DMSO) *δ* 11.87 (br, 1H), 8.70 (br, 1H), 7.91 (t, *J* = 5.4 Hz, 1H), 7.58 (dd, *J* = 10.7, 1.4 Hz, 1H), 7.40 (t, *J* = 7.2 Hz, 1H), 7.36 (d, *J* = 8.7 Hz, 1H), 7.24−7.17 (m, 1H), 6.69−6.64 (m, 1H), 3.93 (s, 2H), 3.58 (s, 2H), 3.41 (t, *J* = 5.8 Hz, 2H), 3.21–3.16 (m, 2H), 2.40 (t, *J* = 6.5 Hz, 2H), 2.31 (t, *J* = 6.4 Hz, 2H); ^19^F NMR (376 MHz, DMSO) *δ *−129.10, −134.05 (d, *J* = 18.2 Hz), −145.19 (d, *J* = 19.1 Hz); HRMS (ESI) calcd. for C_21_H_22_F_3_IN_3_O_6_ [M+H]^+^ 596.0505; found, 596.0482.

#### 3.2.7. General Procedure for the Synthesis of 2,5-Dioxopyrrolidin-1-yl4-((3-(3,4- difluoro-2-((2-fluoro-4-iodophenylamino)benzamido)propyl)amino)-4-oxobutanoate **10**

To a solution of 4-((3-((3,4-difluoro-2-((2-fluoro-4-iodophenyl)amino)benzamido)oxy)propyl) amino)-4-oxobutanoic acid **8b** (282 mg, 0.5 mmol) in 5 mL of (DMF) were added NHS (0.126 g, 1.1 mmol) and DCC (0.226 g, 1.1 mmol). The resulting mixture was stirred at room temperature for 10 h. The dicyclohexylurea (DCU) by-product was filtered off. The filtrate was evaporated to dryness under vacuum to give a crude product, which was then taken up in 1 mL of CH_2_Cl_2_. The insoluble solid was filtered off. The filtrate was concentrated and purified by column chromatography to give the product **10** as a white solid (281 mg, yield 85%); mp. 103.5−104.5 °C; IR (neat) 1737, 1653, 1590, 1497, 1496; ^1^H NMR (400 MHz, CDCl_3_) *δ* 10.24 (br, 1H), 7.84 (br, 1H), 7.44−7.38 (m, 2H), 7.31 (d, *J* = 8.5 Hz, 1H), 6.96−6.90 (m, 1H), 6.56−6.51 (m, 1H), 3.96 (t, *J* = 5.4 Hz, 2H), 3.49 (q, *J* = 5.6 Hz, 2H), 3.01 (t, *J* = 6.7 Hz, 2H), 2.82 (s, 4H), 2.76 (t, *J* = 6.7 Hz, 2H), 1.85-1.79(m, 2H); ^19^F NMR (376 MHz, CDCl_3_) *δ* −128.49, −129.02 (d, *J* = 19.2Hz), −142.57 (d, *J* = 19.5Hz); HRMS (ESI) calcd. for C_24_H_22_F_3_IKN_4_O_6_ [M+K]^+^ 685.0173; found, 685.0355.

#### 3.2.8. General Procedure for the Synthesis of Conjugate **9a** and **9b−e**

A solution of **8a** (2.2 mg, 4 μmol), PyBOP (2.1 mg, 4 μmol), diisopropylethylamine (0.52 mg, 4 μmol) was stirred for 30 min at 0 °C. Then the cyclo(RGDyK) (1.2 mg, 2 μmol) was added to the solution and stirred until the peptide was completely disappeared. The desired product was isolated by semi-preparative HPLC. The collected fractions were combined and lyophilized to give a fluffy white powder **9a**. The yield was 1.0 mg (45%); HRMS (ESI) calcd for C_46_H_55_F_3_IN_12_O_12_ [M-H]^+^ 1151.3059; found, 1151.3623. The conjugate **9a** was purified on a LC3000 HPLC system with a UV3000 UV-VIS Detector (CXTH) using a Daisogel C-18, 30 × 250 mm, 10 µm, 100 Å column. Elution was performed by a linear gradient with an increase of 2.25% acetonitrile per minute in the presence of 0.1% TFA (20 mL/min, 254 nm). The purity of the conjugate **9a** was confirmed by the same gradient system using a Gemini C-18, 10 × 250 mm, 5µm, 110 Å column with a flow of 2 mL/min; Rt = 34.0 min; purity = 98.3%.

**9b** was obtained in 50% yield by using the same method of **9a**. HRMS (ESI) calcd for C_47_H_59_F_3_IN_12_O_12_ [M+H]^+^ 1167.3372; found, 1167.04; The purity of the conjugate **9b** was confirmed by using the same HPLC procedure; Rt = 34.2 min; purity = 97.9%.

**9c** was obtained in 52% yield by using the same method of **9a**. HRMS (ESI) calcd for C_48_H_62_F_3_IN_12_O_12_ [M+2H]^+^ 1182.36; found, 1182.15; The purity of the conjugate **9c** was confirmed by using the same HPLC procedure; Rt =34.5 min; purity = 95.7%.

**9d** was obtained in 60% yield by using the same method of **9a**. HRMS (ESI) calcd for C_49_H_63_F_3_IN_12_O_12_ [M+2H]^+^ 1196.38; found, 1196.11; The purity of the conjugate **9d** was confirmed by using the same HPLC procedure; Rt = 34.4 min; purity = 95.4%.

**9e** was obtained in 42% yield by using the same method of **9a**. HRMS (ESI) calcd for C_48_H_60_F_3_IN_12_O_13_ [M]^+^ 1196.3400; found, 1196.7208; The purity of the conjugate **9e** was confirmed by using the same HPLC procedure; Rt = 34.2 min; purity = 97.5%.

#### 3.2.9. General Procedure for the Synthesis of Conjugates **9f–h**

To a solution of the 2,5-dioxopyrrolidin-1-yl4-((3-(3,4-difluoro-2-((2-fluoro-4-iodophenyl)amino) benzamido)propyl)amino)-4-oxobutanoateactivated ester**10** (4.05 mg, 3 μmol) in anhydrous DMF (1.5 mL) was added the cyclic RGD peptide dimer E[c(RGDyK)]_2_ (4.0 mg, 6 μmol). The pH of the resulting mixture was adjusted to 8.5–9.0 with diisopropylethyl amine (DIPEA). The reaction was stirred at room temperature overnight and the desired product was isolated by semi-preparative HPLC. The collected fractions were combined and lyophilized to give a fluffy white powder **9f** (2.89 mg, yield 50%); HRMS (ESI) calcd. for C_79_H_105_F_3_IN_22_O_22_ [M+H]^+^ 1897.6771; found, 1897.8009; The purity of the conjugate **9f** was confirmed by using the same HPLC procedure of **9a**; Rt = 31.7 min; purity = 96.8%.

**9g** was obtained in 70% yield by using the same method of **9f**. HRMS (ESI) calcd. for C_59_H_82_N_12_F_3_IO_17_ [M+H]^+^ 1414.49; found, 1414.38; The purity of the conjugate **9g** was confirmed by using the same HPLC procedure; Rt = 34.2 min; purity = 98.5%.

**9h** was obtained in 45% yield by using the same method of **9f**. HRMS (ESI) calcd. for C_48_H_61_F_3_IN_12_O_12_ [M+H]^+^ 1181.35, found, 1181.51; The purity of the conjugate **9h** was confirmed by using the same HPLC procedure; Rt = 34.1 min; purity = 98.3%.

#### 3.2.10. General Procedure for the Synthesis of (S)-4-(3-((3,4-difluoro-2-((2-fluoro-4-iodophenyl) amino)benzamido)oxy)-2-hydroxypropoxy)-4-oxobutanoic Acid **11**

**11** was obtained through the method similar to compound **8a** as a white solid(91% yield); mp. 64.1−66.7 °C; IR (neat) 2926, 1732, 1602, 1496, 1280, 1166, 831; ^1^H NMR (400 MHz, CDCl_3_) *δ* 16.13 (s, 1H), 8.12 (br, 1 H), 7.37 (d, *J* = 10.0 Hz, 2H), 7.29 (d, *J* = 8.5 Hz, 1H), 6.89−6.82 (m, 1H), 6.56−6.51 (m, 1H), 4.30−3.76 (m, 5H), 2.65 (s, 4H); ^19^F NMR (376 MHz, CDCl_3_) *δ* −127.91, −129.10, −142.65; HRMS (ESI) calcd. for C_20_H_19_F_3_IN_2_O_7_ [M+H]^+^ 583.0189; found, 583.0187.

#### 3.2.11. General Procedure for the Synthesis of (S)-3-((3,4-difluoro-2-((2-fluoro-4-iodophenyl)amino) benzamido)oxy)-2-hydroxypropyl (2,5-dioxopyrrolidin-1-yl) succinate **12**

**12** was obtained through the method similar to compound **10** as a white solid (35% yield); mp. 76.1−78.2 °C; IR (neat) 2926, 1737, 1516, 1496, 1274, 1205, 1070, 763, 750; ^1^H NMR (400 MHz, CDCl_3_) *δ* 9.83 (s, 1H), 8.26 (br,1H), 7.40−7.37 (m, 2H), 7.30 (d, *J* = 8.5Hz), 6.88−6.82 (m, 1H), 6.58−6.53(m, 1H), 4.23−3.72(m, 5H), 2.96−2.92(m, 2H), 2.82 (s, 4H), 2.78−2.75(m, 2H); ^19^F NMR (376 MHz, CDCl_3_) *δ* −128.14 (d, *J* = 4.4Hz), −128.98 (d, *J* = 3.2Hz), −143.15 (d, *J* = 20.9Hz); HRMS (ESI) calcd. for C_24_H_22_F_3_IN_3_O_9_ [M+H]^+^ 680.0353; found, 680.0338.

#### 3.2.12. General Procedure for the Synthesis of Conjugate **13**

**13** was obtained through the method similar to compound **9f** as a white soild (40% yield); HRMS (ESI) calcd for C_47_H_59_F_3_IN_12_O_13_ [M+H]^+^ 1184.3162; found, 1184.1226. The purity of the conjugate **13** was confirmed by the same HPLC procedure; Rt = 34.2 min; purity = 95.7%.

### 3.3. General Procedure for the HTRF Phosphor-MEK1 and HTRF Phosphor-ERK2 Assays

The HTRF phosphor-MEK1 assay was used to test for capability of compounds to inhibit the phosphorylation reaction of inactive MEK1. The assay was performed under the following reaction conditions: a constitutively active BRAF was obtained from Carna Biosciences (Kobe, Japan) and it was tested at a concentration of 7 nM.The test compounds dissolved in DMSO at 10 mM were diluted in assay buffer (50 mM HEPES buffer, pH 7.0, 10 mM MgCl_2_, 1 mM DTT, 0.5 mM or thovanadate, and 0.01% BSA) and added to 40 nMGST-labeled inactive MEK1, and the reaction was initiated with 100 μM ATP. Phosphorylated MEK1 was determined quantitatively by formation of a complex with specific Eu-labeled anti-phospho p44/42 MAPK (Thr202/Tyr204) antibody and anti-GST-XL665.After 3 h, time-resolved fluorescence was read using a FlexStation 3 plate reader (Molecular Devices, Sunnyvale, CA, USA). Excitation of europium (the donor) using a 314 nm excitation filter results in energy transfer to the fluorophore of anti-GST-XL665 which is detected by an increase in the fluorescence emission of anti-GST-XL665 at 668 nm and a decrease in the fluorescence emission of europium at 620 nm. The curve-fitting software GraphPad Prism 4.0 was used to generate the curves and determine the IC_50_ values. The HTRF phosphor-ERK2assay was used to test for inhibition of compounds relative to active MEK1. The assay was performed under the following reaction conditions: A constitutively active MEK1 was obtained from Carna Biosciences and it was tested at a concentration of 5.72 nM with 40 nM inactive ERK2, 30 μM ATPand test compounds at a variety of concentrationsin assay buffer. After 2 h reaction at RT, the phosphop-ERK2 was terminated by the addition of 5 μL Anti-Phospho p44/42 MAPK (Thr202/Tyr204)-Cryptate(CisBiol) and 26 nM Anti-GST-XL665. The calculation method of HTRF ratio was the same with that in HTRF phosphor-MEK1 assay.

### 3.4. Cell-Based Assays

#### 3.4.1. Cell Culture

A375, U87, A549 cell line, which were obtained from the National Platform of Experimental Cell Resources for Sci-Tech (Beijing, China), were cultured in Dulbecco’s modified Eagle’s medium (DMEM; Hyclone, Logan, UT, USA), supplemented with 10% heat-inactivated FBS (Hyclone), 100 U/mL penicillin, and 100 μg/mL streptomycin (Wako Pure Chemical Industries, Ltd., Osaka, Japan). The cells were cultured in a humidified atmosphere of 5% CO_2_ in air at 37 °C.

#### 3.4.2. Anti-Proliferation Assay

The cytotoxicity of the RGD-MKE1 conjugates on A375 melanoma cells, ERK signal pathway hyper-activated cell line, was studied *in vitro* by using an SRB assay. A375 cells (4.5 × 10^3^) or U87 cells (5.0 × 10^3^) were plated in each well of a 96-well tissue culture plate. Medium supplemented with 10% FBS was added, and cells were allowed to adhere for 24h. Cells were then incubated with serial dilutions of PD0325901 and RGD-MKE1 conjugates for 72 h in triplicate. Then the medium was removed from the 96-well plate, and 10% ice-cold trichloroacetic acid (TCA, 200 μL) was added. The plate was kept at 4 for one hour after which was washed five times with deionized water, then stained with Sulforhodamine B (SRB, 100 μL, Sigma, St. Louis, MO, USA) before air drying. After washing with 1% acetic acid, the bound dye was solubilized with Tris base A (Sigma) and 100 μL of each sample were transferred into a 96-well plate, and then absorbance was read in a 96-well plate reader at 540 nm.Inhibitory ratio (%) = [(OD_control_ − OD_treated_)/(OD_control_)] × 100%. Cytotoxicity was also expressed as the concentration of compounds inhibiting cell growth by 50% (IC_50_ value) which was calculated by the GraphPad Prism 5 software.

#### 3.4.3. Western Blotting Assay

To evaluate the expression levels of ERK singal pathway after treated with conjugates, A375 cells were treated with compounds for 2 h respectively. Confluent cells were washed with ice-cold phosphate-buffered saline (PBS), and treated with lysis buffer (Beyotime Institute of Biotechnology, Suzhou, China) on ice for 1 h. Then the lysates were collected by scraping from the plates and then centrifuged at 13,000 rpm at 4 °C for 15 min. The protein concentration was quantitated using a Pierce BCA Protein Assay Kit (Thermo Scientific, Rockford, IL, USA) ona FlexStation 3 Benchtop Multi-Mode Microplate Reader at 562 nm (Molecular Devices). An equal amount of proteins (10 μg per lane) for each sample were analyzed by SDS-PAGE on a 10% gel. After electro-blotting to PVDF membrane (Millipore, Billerica, MA, USA) at 400 mA for 1 h, membranes were blocked with 5% nonfat dry milk for 1 h at room temperature. Membrane were washed with 0.1% PBST three times and then incubated with primary antibodies overnight at 4 °C. Antibodies against pERK and ERK (Cell Signaling Technology, Inc., Danvers, MA, USA) were utilized as the primary antibodies. After removal of the primary antibody, the membranes were washed with PBST and incubated for 3 h at room temperature with a secondary horseradish peroxidase-coupled IgG antibody (Cell Signaling Technology, Inc.). Finally, protein products were analyzedby Image Lab^TM^ software(ChemiDoc^TM^ XRS System, BIO-RAD, Hercules, CA, USA) with. Expression levels of pERK and ERK protein were normalized against β-actin protein expression levels.

#### 3.4.4. Flow Cytometric Cell Cycle Analysis

The proportion of A375 cells in different phases were measured by flow cytometry analysis. A375 cells were seeded in 6-well plates (3 × 10^6^ cells/well) at 37 °C under an atmosphere of 5% CO_2_ for 24 h. After 24 h incubation with compounds, the cells were collected and washed twice with PBS. Each group was fixed overnight in ice-cold 70% ethanol at 4 °C and re-suspended in PBS containing 0.1 mg/mL RNase A and 5 ug/mL propidiumidodide (PI). After incubated for 30min at room temperature in the dark, the cells were analyzed by flow cytometry using a FACS Calibur instrument (Becton-Dickinson, Franklin Lakes, NJ, USA). Here the untreated cells were used as the control. The analysis of cell cycle distribution was subsequently performed using the Modfit software.

#### 3.4.5. DNA Replication Study

Proliferating A375 cells were determined by the EdU In Vitro Imaging Kit (Ribobio, Guangzhou, China).EdU (5-ethynyl-2'-deoxyuridine) is a nucleoside analog of thymidine that is incorporated into DNA during active DNA synthesis only by proliferating cells. After incorporation, a fluorescent molecule was added that reacted specifically with EdU, making possible fluorescent visualization of proliferating cells. According to the manufacturer’s protocol, A375 cells were cultured in 96-well plates at 4.5 × 10^3^cells per well and left to grow at 37 °C under an atmosphere of 5% CO_2_ 24 h. Cells were treated with the compounds of 1 μM for 24 h. After that, cells were incubated with 50 μMEdU for 2 h at 37 °C, and then were fixated with 4% paraformaldehyde for 15 min. The cells were stained by 1× Apollo staining fluid (included in the kit) before permeabilization with 0.5% TritonX-100. Cell nucleuses were stained with 1× Hoechst 33342 for 30 min.The cells were tested by the OLYMPUS IX81 inverted fluorescence microscope (Olympus America Inc., Center Valley, PA, USA). The EdU^+^ cells were detected at the excitation of 550 nm. The EdU^−^ cells were detected at the excitation of 350 nm.

## 4. Conclusions

We have designed, synthesized and biologically evaluated two novel series of RGD-MEKI conjugates based on the MEK1/2 inhibitor PD0325901 for specific integrin receptor-targeting anti-cancer therapy. The biological analysis showed that all the conjugates efficiently inhibited ERK pathway and arrested melanoma A375 cells at G1 phase with the same drug mechanism as the parent drugPD0325901. The anti-proliferation activity of conjugates was inhibited by the scrambled peptide conjugate **9h**, which indicated that the RGD-MEKI conjugates underwent an integrin receptor mediated route. Compared to the alkoxylamine analog conjugates **9a**–**g**, direct conjugate RGD-PD0325901 (**13**) exhibited excellent anti-tumor properties. Future reports will describe modifications of this conjugating drug candidate for optimization of *in vivo* properties. We believe that these preliminary studies lay a foundation that may be built upon for a new tumor targeting therapy based on small molecule targeted drugs.

## Abbreviations

MEK: mitogen-activated protein kinase/extracellular signal regulated kinase
ERK: mitogen-activated protein kinase/extracellular signal-regulated kinase
pERK: phosphor-ERK; HTRF, homogeneous time-resolved fluorescence
NSCLC: non-small cell lung cancer; SRB, sulforhodamine B
PyBOP: benzotriazole-1-yl-oxy-*tris*-pyrrolidino-phosphonium hexafluoro phosphate
NHS: *N*-hydroxy succinimide
DCC: *N, N'*-dicyclohexyl-carbodiimide
CH_2_Cl_2_: methylene chloride
FBS: fetal bovine serum
BCA: bicinchoninic acid
SDS-PAGE: sodium dodecyl sulfate polyacrylamide gel electrophoresis
PVDF membrane: polyvinylidenedifluoride membrane
PBST: phosphate buffered saline Tween-20

## Supplementary Materials

Supplementary materials can be accessed at: http://www.mdpi.com/1420-3049/18/11/13957/s1.
