# Supplementary material for: Synthesis and Biological Evaluation of RGD-Conjugated MEK1/2 Kinase Inhibitors for Integrin-Targeted Cancer Therapy
Source: Molecules. 2013 Nov 12;18(11):13957–78. doi: 10.3390/molecules181113957 (PMC6269693; doi:10.3390/molecules181113957)
Supplement: Supplementary file 1 [file molecules-18-13957-s001.pdf]

Supplementary Materials

1. HRMS Spectrogram

Figure S1. MS spectrogram of conjugate **9a**.

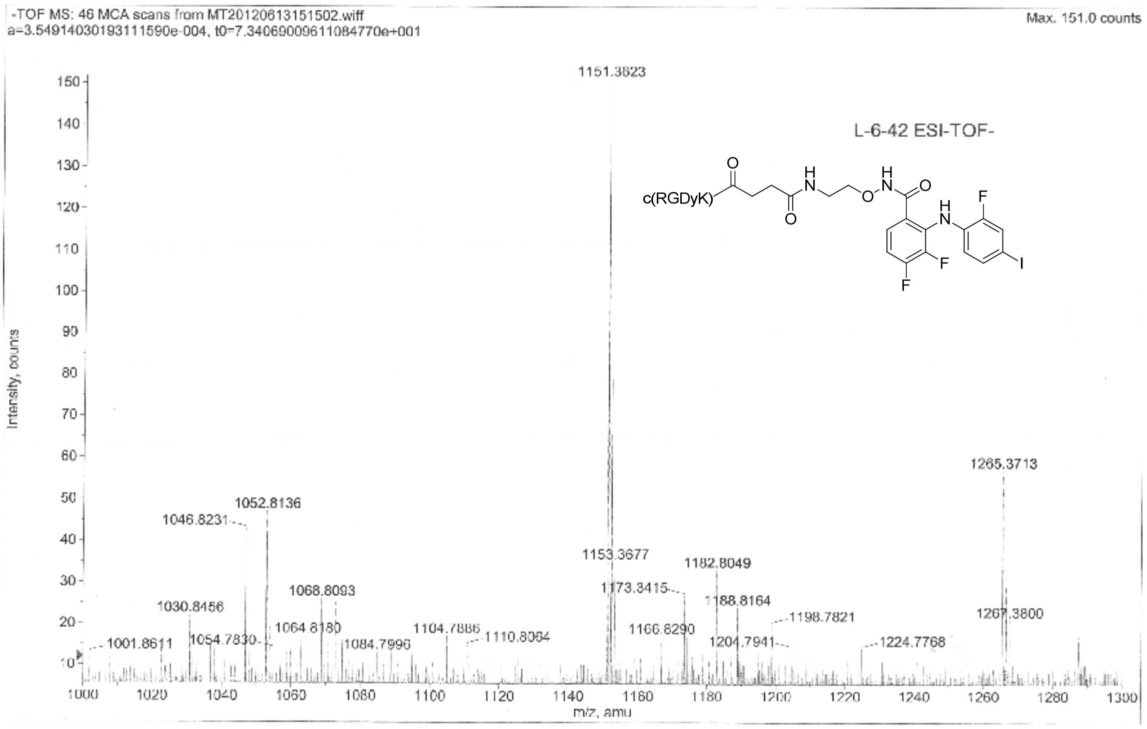

Figure S2. MS spectrogram of conjugate **9b**.

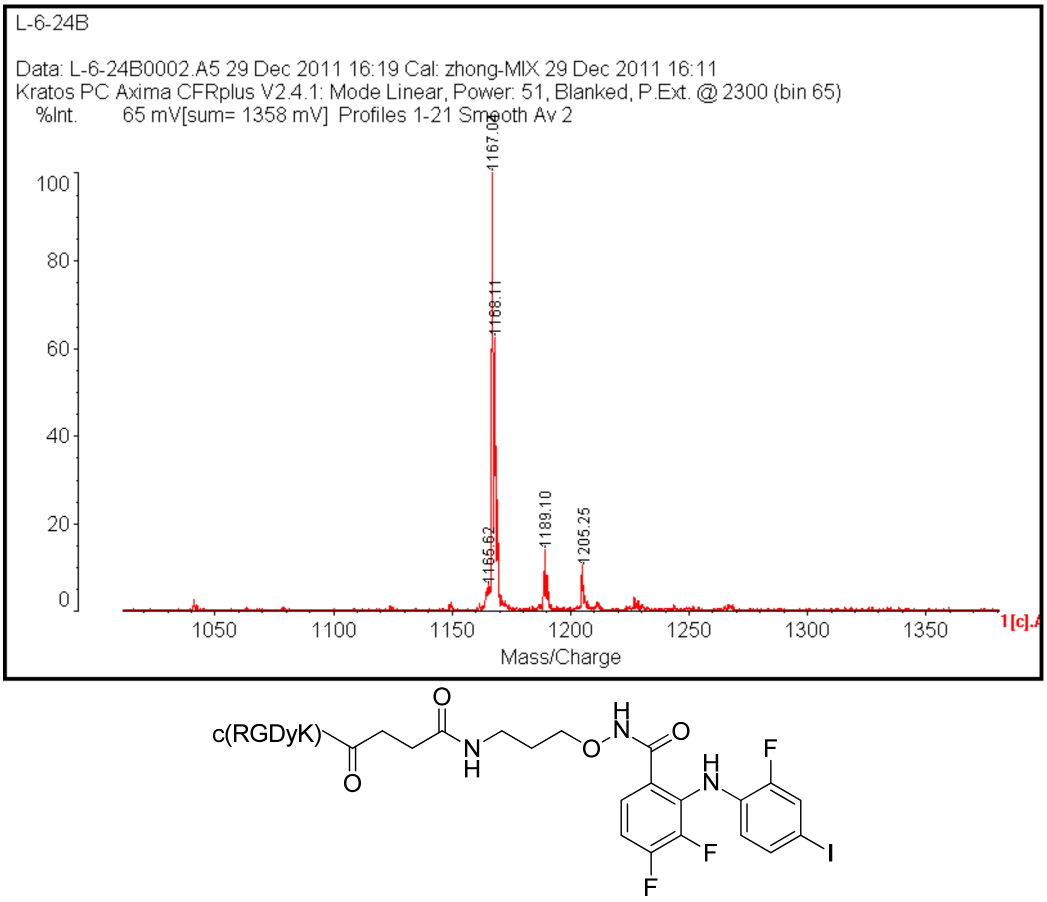

Figure S3. MS spectrogram of conjugate **9c**.

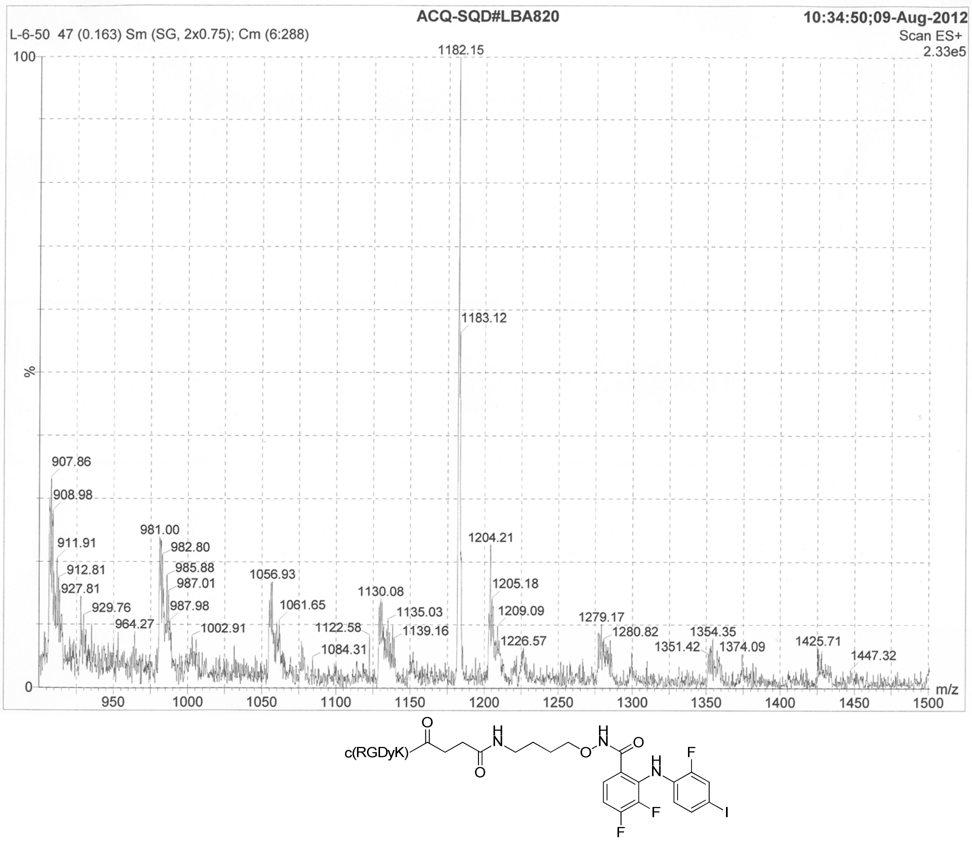

Figure S4. MS spectrogram of conjugate **9d**.

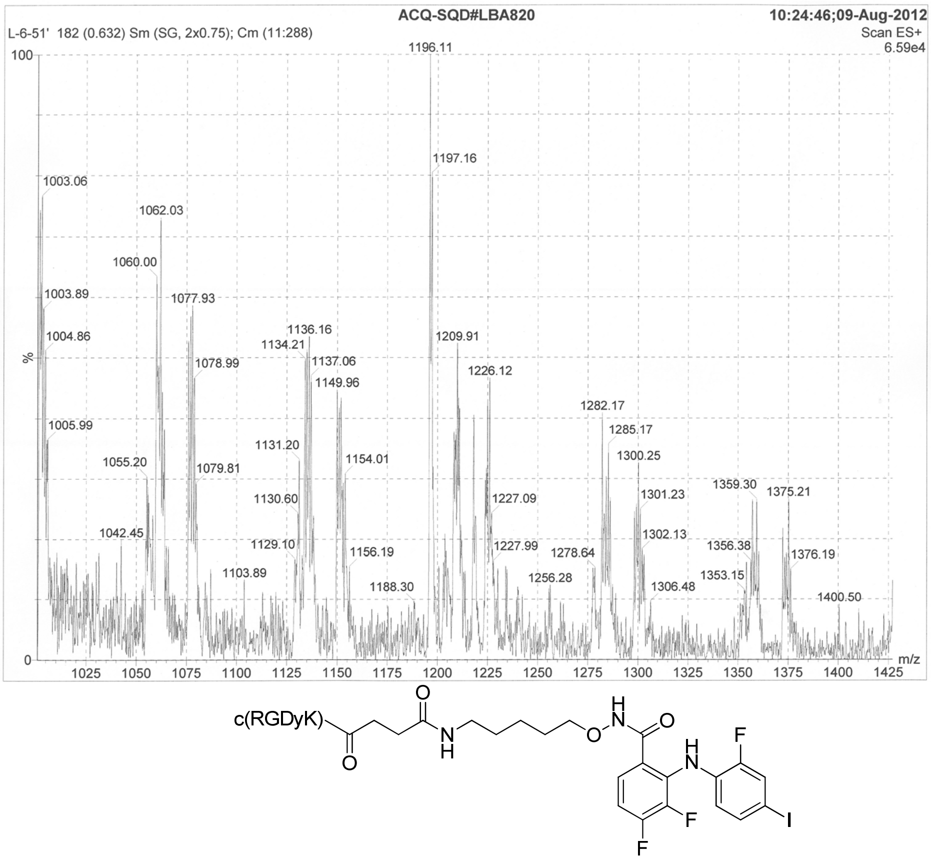

Figure S5. MS spectrogram of conjugate **9e**.

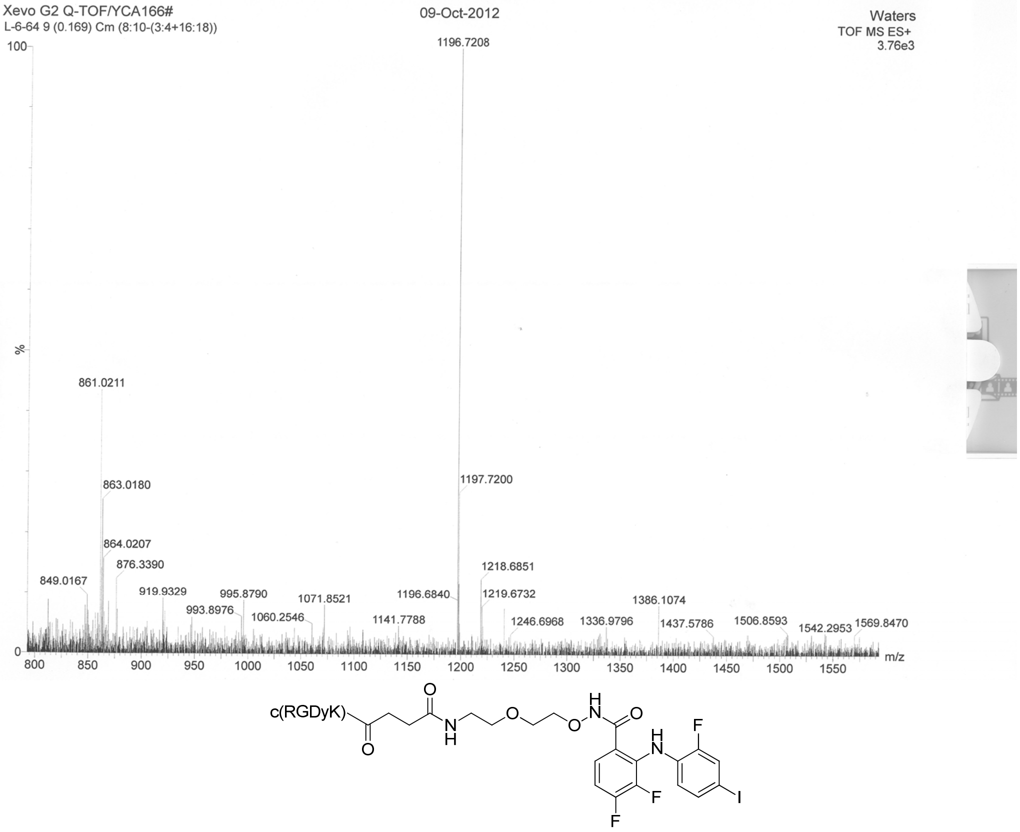

Figure S6. MS spectrogram of conjugate **9f**.

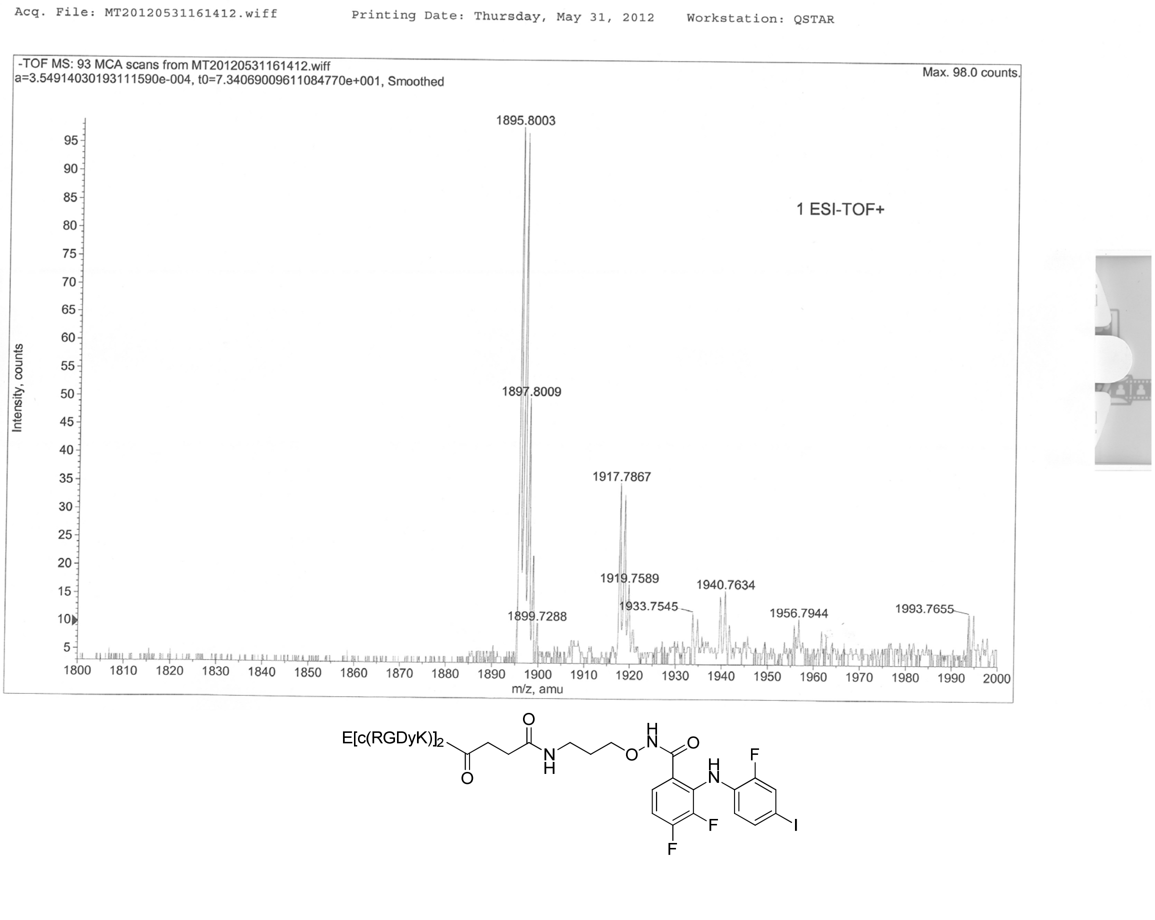

Figure S7. MS spectrogram of conjugate **9g**.

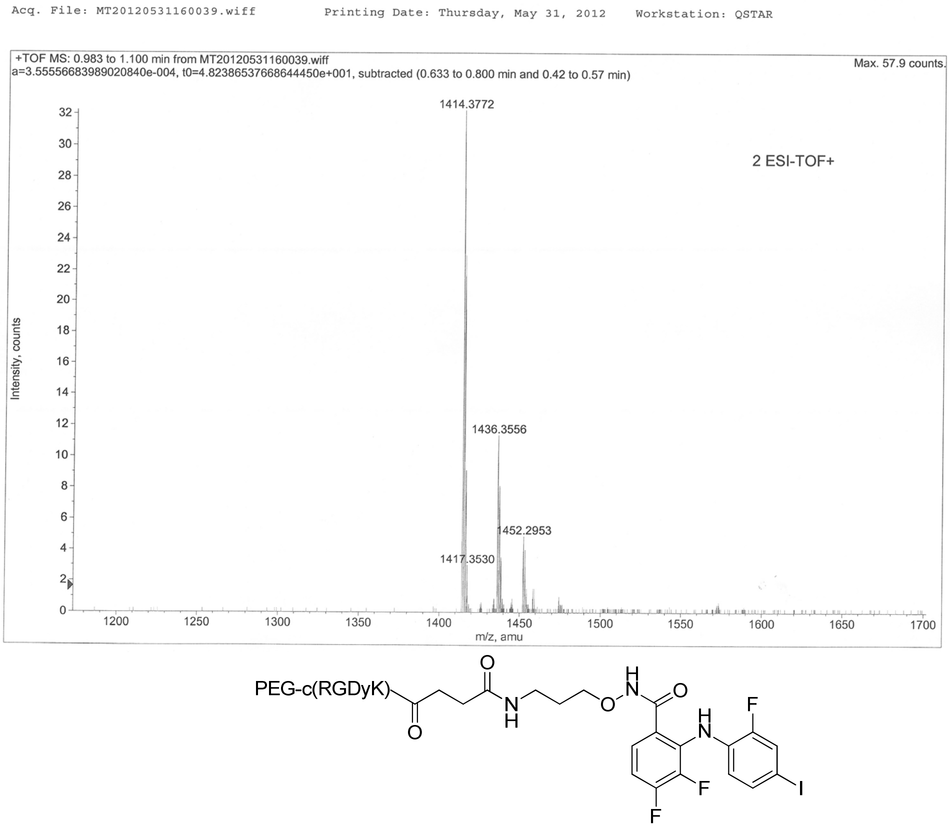

Figure S8. MS spectrogram of conjugate **9h**.

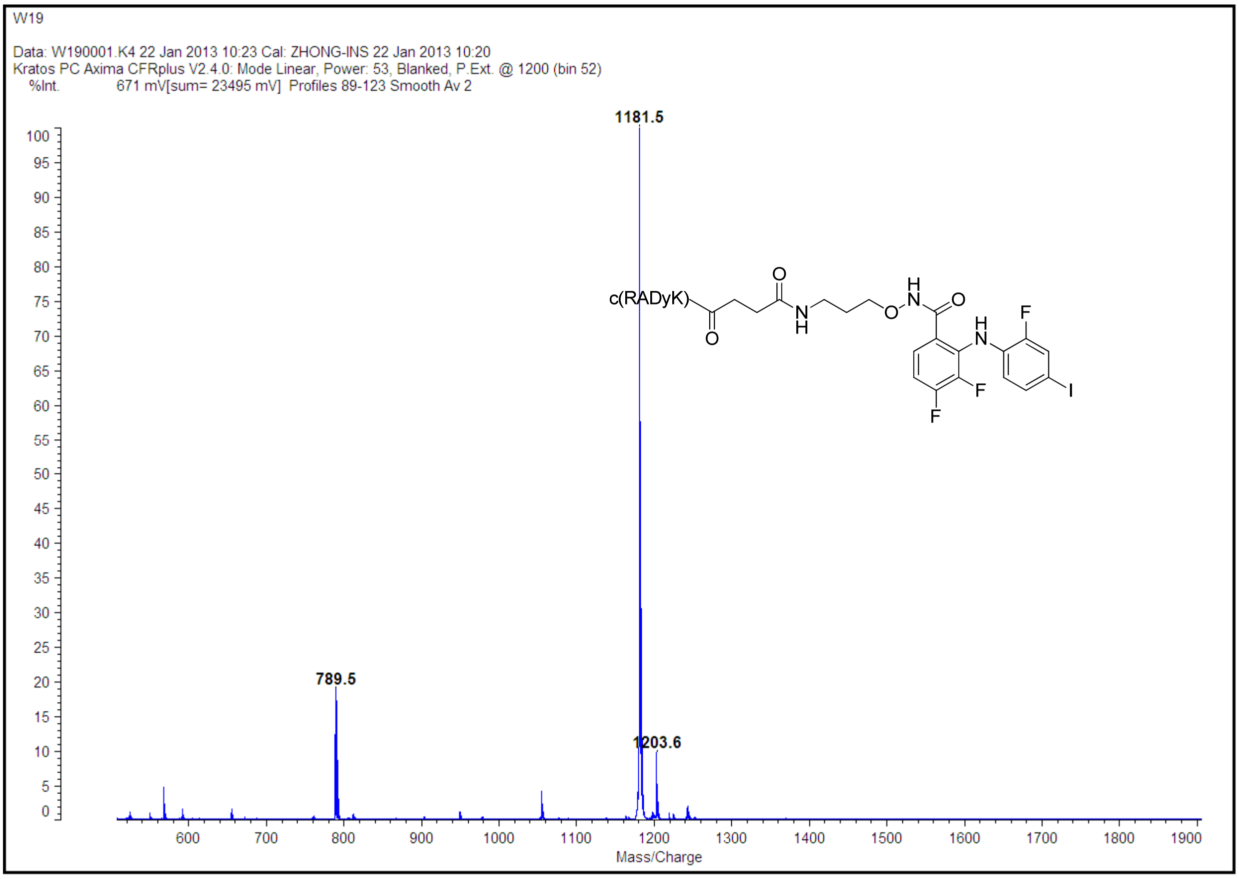

Figure S9. MS spectrogram of conjugate **13**.

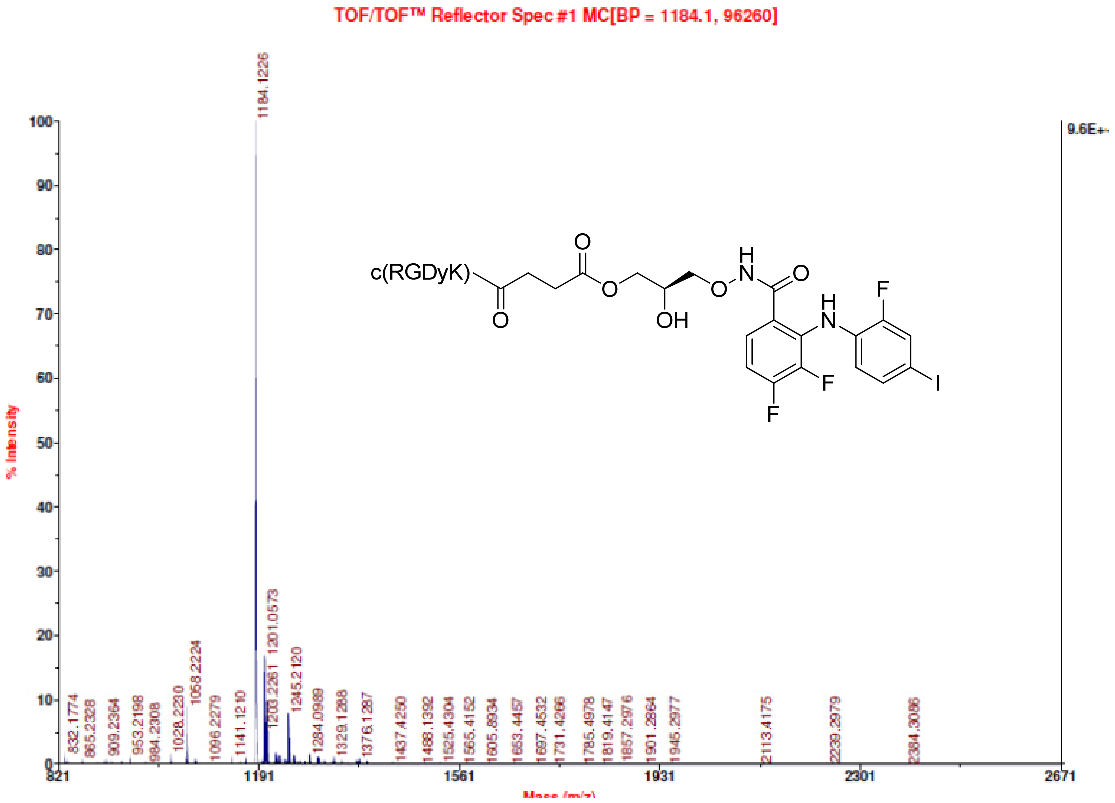

2. HPLC Data

2.1. HPLC Data of Conjugate **9a**

Sample: **9a**
HPLC Column; Gemini 5 μm C18 110A column (5 micron, 250 × 10 mm)
Flow: 2 mL/min
Linear Gradient: 2.25% CH_3CN increased per minute
Wavelength: 254 nm
File opened: E:\data\lixiaoxiao\purity\L-6-107-chundu2(00002,21;39;36).hw, where

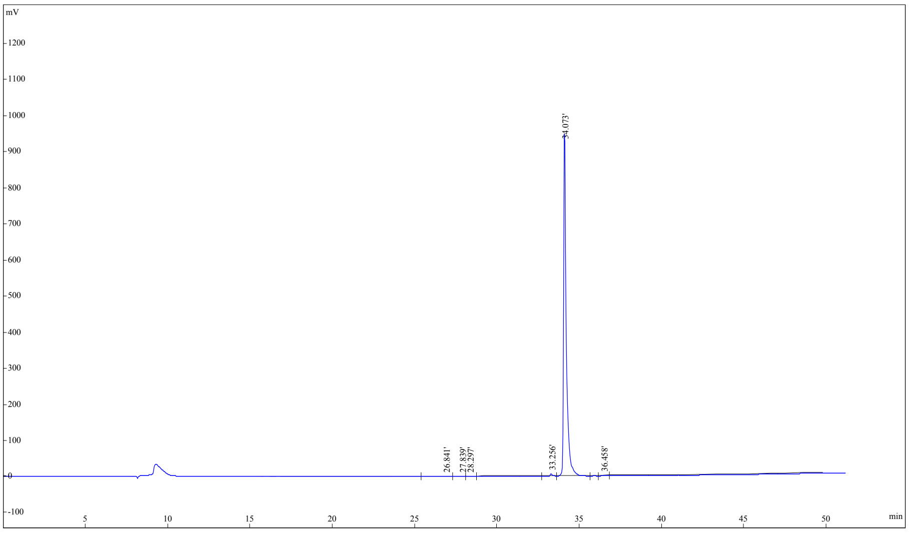

Rank	Time	Name	Conc	Area
1	26.841		0.2299	25531
2	27.839		0.3214	35698
3	28.297		0.2595	28825
4	33.256		0.7134	79234
5	34.073		98.29	10916350
6	36.458		0.1932	21463
Total			100	11107101

purity = 98.3%.

2.2. HPLC Data of Conjugate **9b**

Sample: **9b**

HPLC Column; Gemini 5 µm C18 110A column (5 micron, 250 × 10 mm)

Flow: 2 mL/min

Linear Gradient: 2.25% CH_3CN increased per minute

Wavelength: 254 nm

File Opened: E:\data\lixiaoxiao\purity\L-6-103A-chundu-3(00001,20;40;55).hw, where

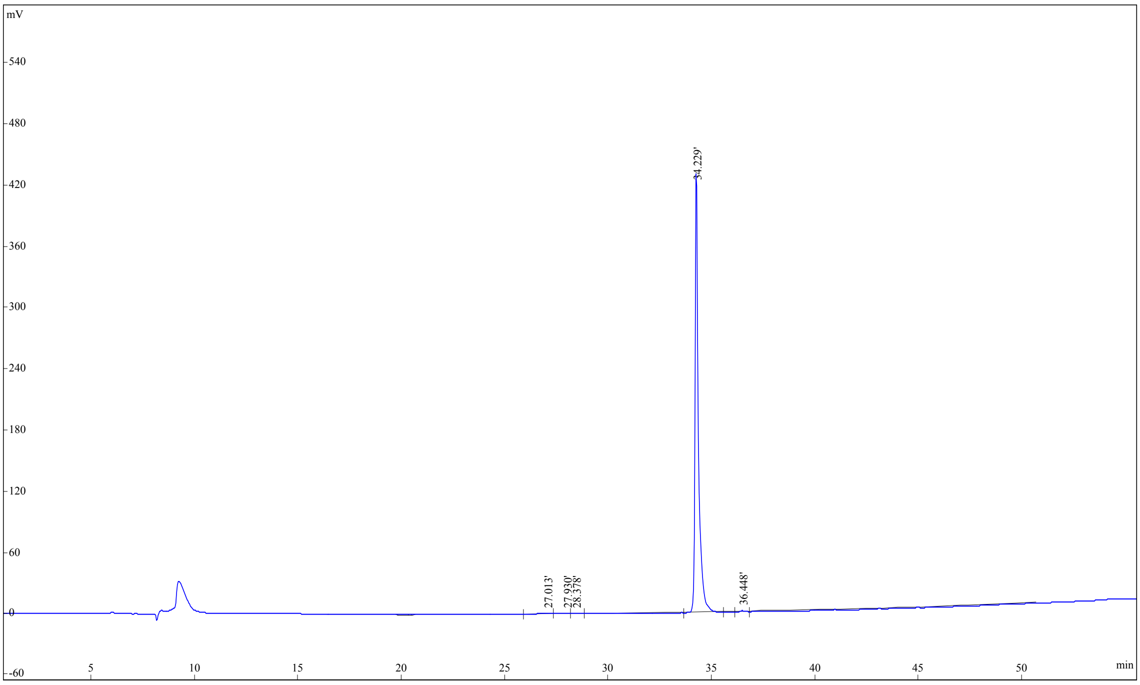

Rank	Time	Name	Conc	Area
1	27.013		0.4343	21965
2	27.930		0.6705	33915
3	28.378		0.5594	28294
4	34.229		97.91	4952175
5	36.448		0.4329	21895
Total			100	505824

purity = 97.9%.

2.3. HPLC Data of Conjugate **9c**

Sample: **9c**
HPLC Column; Gemini 5 µm C18 110A column (5 micron, 250 × 10 mm)
Flow: 2 mL/min
Linear Gradient: 2.25% CH_3CN increased per minute
Wavelength: 254 nm
File opened: E:\data\lixiaoxiao\purity\L-6-106-chundu2-1(00001,12;47;45).hw, where

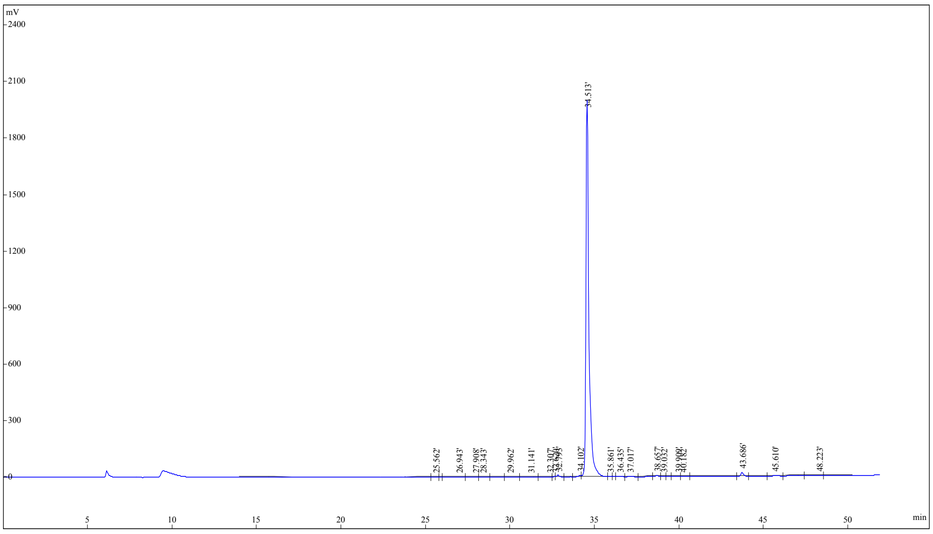

Rank	Time	Name	Conc	Area
1	25.562		0.07965	20737
2	26.943		0.1921	50013
3	27.908		0.2548	66345
4	28.343		0.2054	53469
5	29.962		0.09661	25152
6	31.141		0.1335	34755
7	32.307		0.2049	53344
8	32.620		0.1165	30335
9	32.793		0.3967	103281
10	34.102		0.444	115582
11	34.513		95.74	24925901
12	35.861		0.1066	27750
13	36.435		0.1845	48023
14	37.017		0.08039	20929
15	38.657		0.3009	78335
16	39.032		0.08618	22436
17	39.909		0.165	42951
18	40.182		0.08473	22057
19	43.686		0.8744	227629
20	45.610		0.14	36448
21	48.223		0.1113	28965
Total			100	26034437

purity = 95.7%.

2.4. HPLC Data of Conjugate **9d**

Sample: 9d
HPLC Column; Gemini 5 µm C18 110A column (5 micron, 250 × 10 mm)
Flow: 2 mL/min
Linear Gradient: 2.25% CH_3CN increased per minute
Wavelength: 254 nm
File opened: E:\data\lixiaoxiao\purity\L-6-105-chundu(00003,14;47;19).hw, where

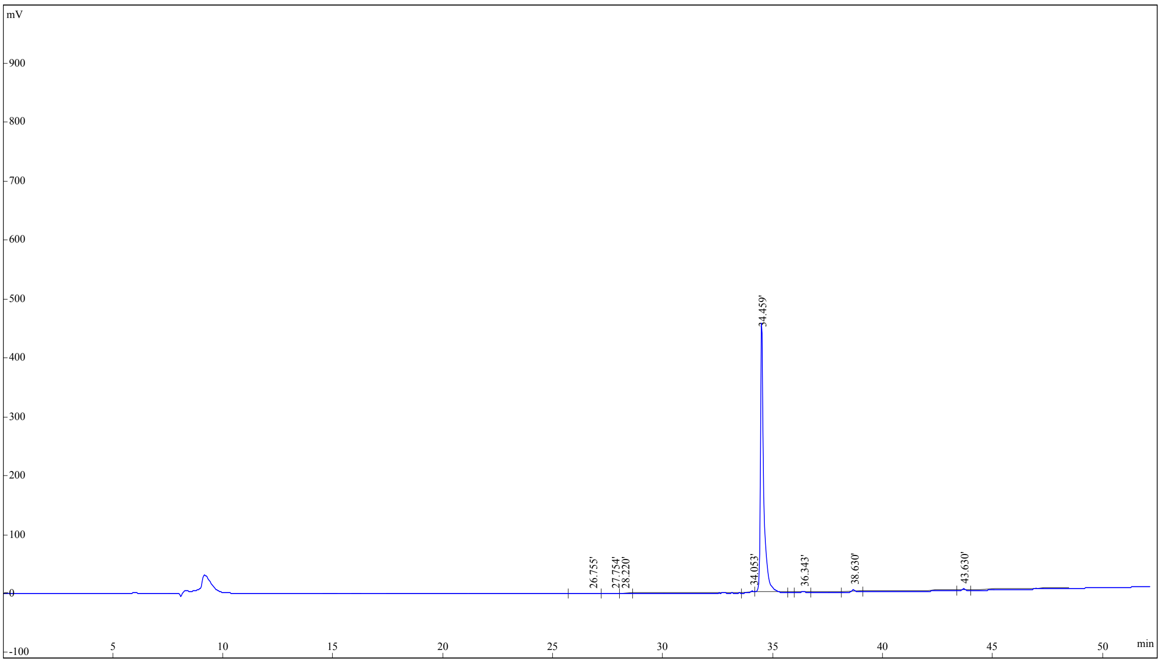

Rank	Time	Name	Conc	Area
1	26.755		0.4843	24620
2	27.754		0.6522	33158
3	28.220		0.533	27095
4	34.053		0.899	45703
5	34.459		95.41	4850458
6	36.343		0.4546	23109
7	38.630		0.8664	44049
8	43.630		0.706	35894
Total			100	5084086

purity = 95.4%.

2.5. HPLC Data of Conjugate **9e**

Sample: **9e**
HPLC Column; Gemini 5 µm C18 110A column (5 micron, 250 × 10 mm)
Flow: 2 mL/min
Linear Gradient: 2.25% CH_3CN increased per minute
Wavelength: 254 nm
File opened: E:\data\lixiaoxiao\purity\L-6-104B-chundu2-2(00004,19;04;01).hw, where

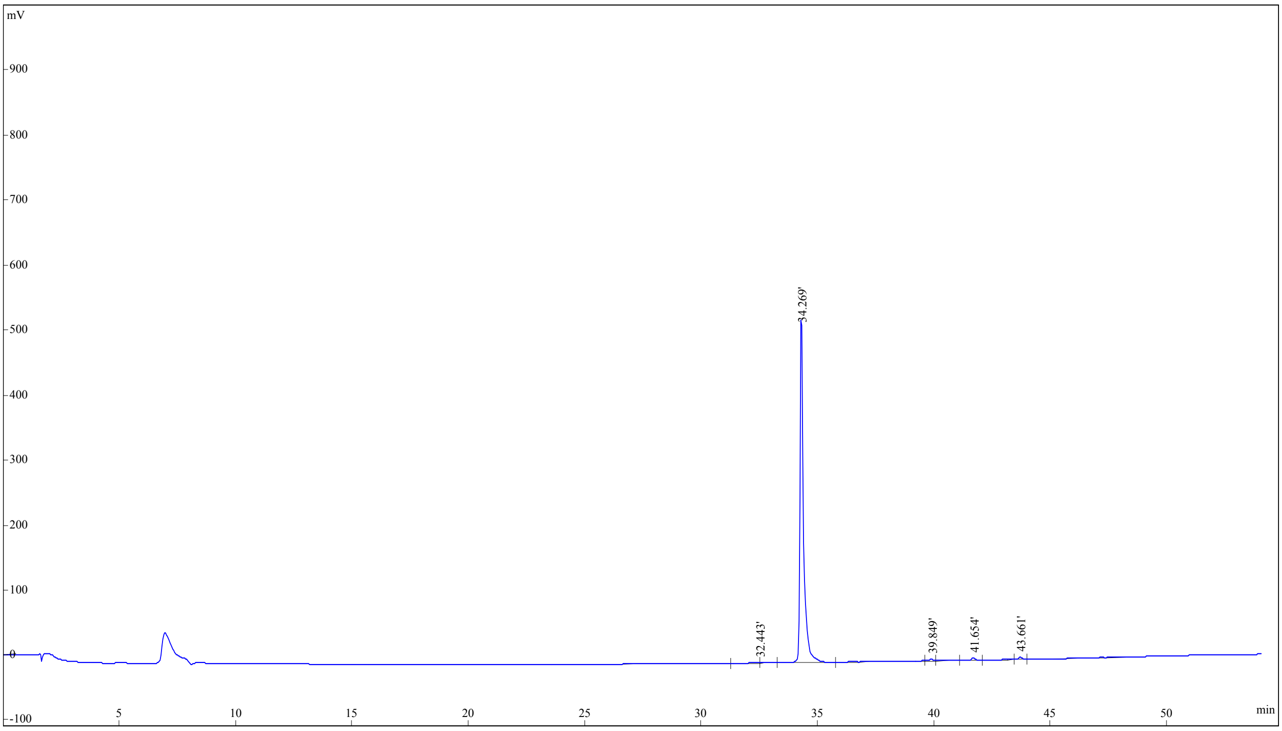

Rank	Time	Name	Conc	Area
1	32.443		0.365	21211
2	34.269		97.54	5668904
3	39.849		0.5991	34817
4	41.654		0.7889	45850
5	43.661		0.7073	41109
Total			100	5811891

purity = 97.5%.

2.6. HPLC Data of Conjugate **9f**

Sample: **9f**
HPLC column: Gemini 5 µm C18 110A column (5 micron, 250 × 10 mm)
Flow: 2 mL/min
Linear Gradient: 2.25% CH_3CN increased per minute
Wavelength: 254 nm
File opened: E:\data\lixiaoxiao\purity\L-6-103B-chundu2(00003,20;07;01).hw, where

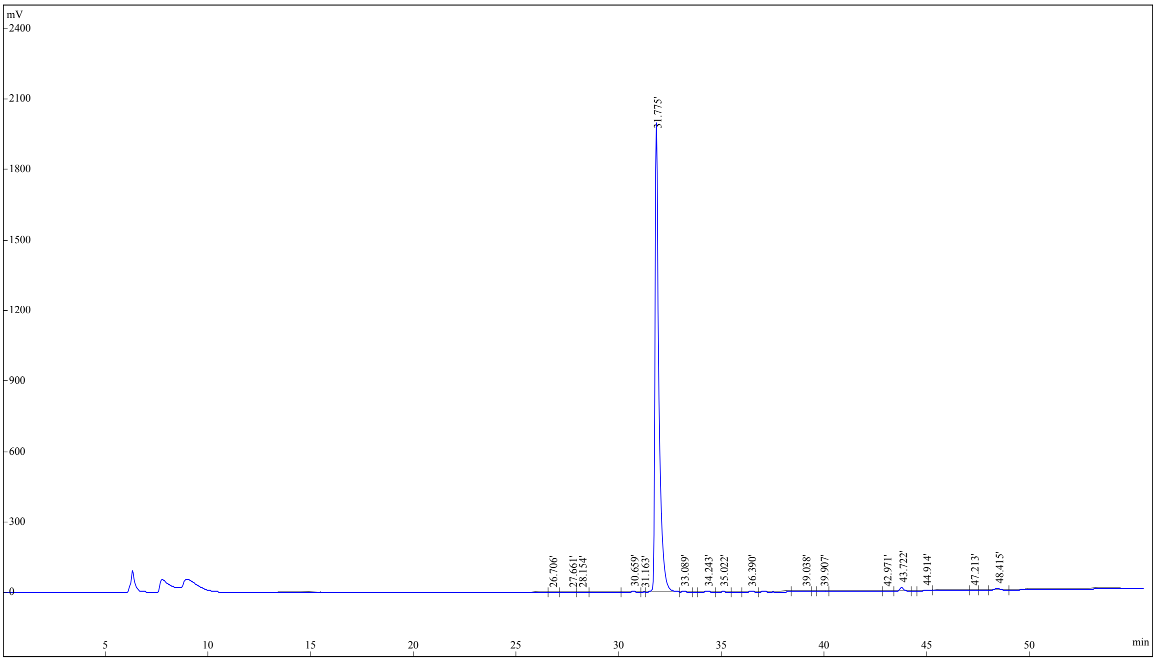

Rank	Time	Name	Conc	Area
1	26.706		0.0891	24687
2	27.661		0.1401	38817
3	28.154		0.103	28543
4	30.659		0.2529	70075
5	31.163		0.09092	25192
6	31.775		96.8	26818893
7	33.089		0.2187	60940
8	34.243		0.22	30335
9	35.022		0.1131	31344
10	36.390		0.09268	25679
11	39.038		0.2183	60470
12	39.907		0.1172	32467
13	42.971		0.1245	34507
14	43.722		0.691	191456
15	44.914		0.1281	35487
16	47.213		0.07504	20791
17	48.415		0.5319	147368
Total			100	27707302

purity = 96.8%.

2.7. HPLC Data of Conjugate **9g**

Sample: **9g**
HPLC Column: Gemini 5 µm C18 110A column (5 micron, 250 × 10 mm)
Flow: 2 mL/min
Linear Gradient: 2.25% CH_3CN increased per minute
Wavelength: 254 nm
File Opened: E:\data\lixiaoxiao\purity\L-6-104A-chundu2-2(00002,16;44;03).hw, where

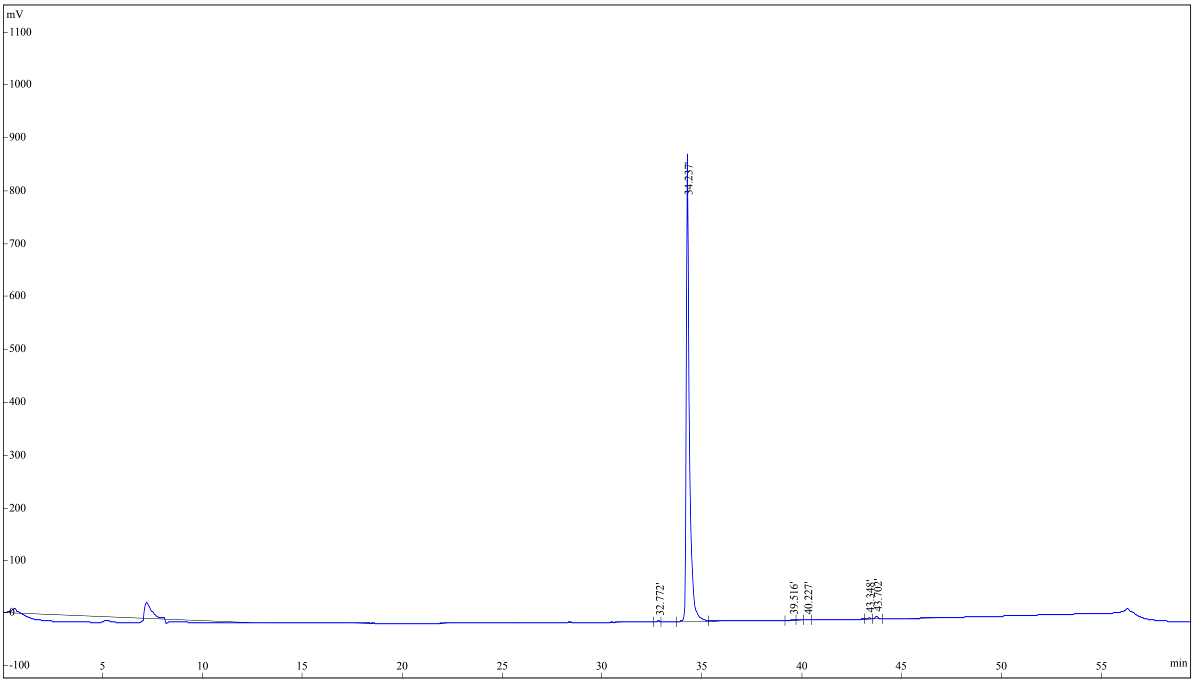

Rank	Time	Name	Conc	Area
1	32.772		0.2435	23583
2	34.237		98.49	9538024
3	39.516		0.2069	20031
4	40.227		0.2148	20805
5	43.348		0.2509	24296
6	43.702		0.5922	57344
Total			100	9684083

purity = 98.5%.

2.8. HPLC Data of Conjugate **9h**

Sample: **9h**
HPLC Column: Gemini 5 µm C18 110A column (5 micron, 250 × 10 mm)
Flow: 2 mL/min
Linear Gradient: 2.25% CH_3CN increased per minute
Wavelength: 254 nm
File opened: E:\data\lixiaoxiao\purity\L-6-99-chundu3(00009,23;49;18).hw, where

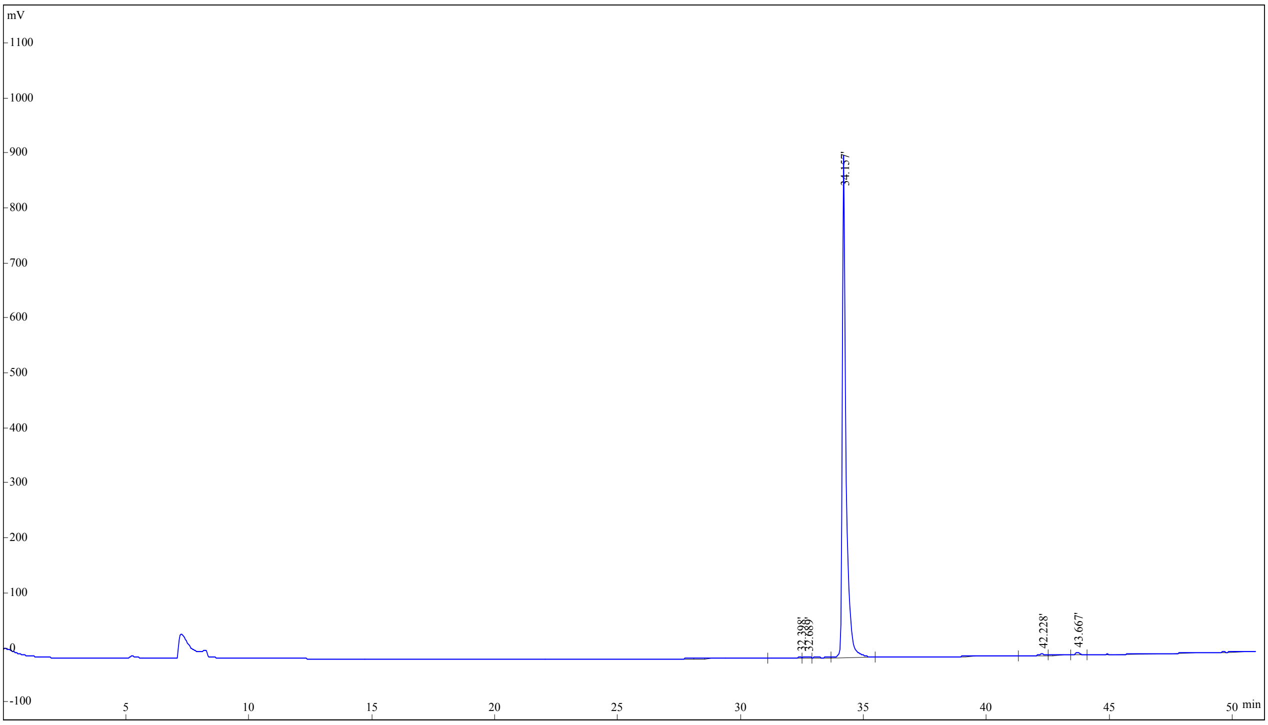

Rank	Time	Name	Conc	Area
1	32.398		0.3225	31858
2	32.689		0.2824	27899
3	34.157		98.31	9711040
4	42.228		0.4565	45096
5	43.667		0.6359	62821
Total			100	9878714

purity = 98.3%.

2.9. HPLC Data of Conjugate **13**

Sample: **13**
HPLC Column: Gemini 5 µm C18 110A column (5 micron, 250 × 10 mm)
Flow: 2 mL/min
Linear Gradient: 2.25% CH_3CN increased per minute
Wavelength: 254 nm
File opened: E:\data\lixiaoxiao\purity\L-6-98-chundu(00008,22;53;34).hw, where

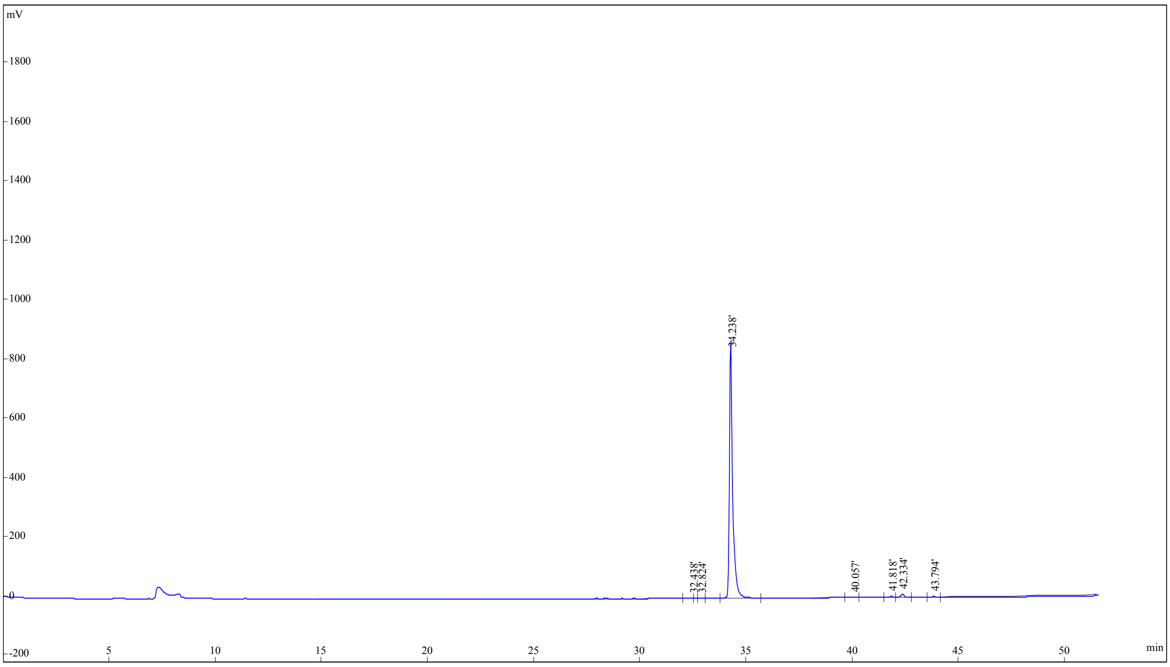

Rank	Time	Name	Conc	Area
1	32.438		0.2352	23424
2	32.824		0.2352	23421
3	34.238		95.68	9527221
4	40.057		0.5703	56784
5	41.818		0.8089	80544
6	42.334		96.8	26818893
7	43.794		0.6252	62256
Total			100	9957941

puryty = 95.7%.
